# Face-to-face panel meetings versus remote evaluation of fellowship applications: simulation study at the Swiss National Science Foundation

**DOI:** 10.1136/bmjopen-2020-047386

**Published:** 2021-05-05

**Authors:** Marco Bieri, Katharina Roser, Rachel Heyard, Matthias Egger

**Affiliations:** 1 Careers Division, Swiss National Science Foundation, Bern, Switzerland; 2 Department of Health Sciences and Medicine, University of Lucerne, Lucerne, Switzerland; 3 Data Team, Swiss National Science Foundation, Bern, Switzerland; 4 Institute of Social & Preventive Medicine, University of Bern, Bern, Switzerland; 5 Population Health Sciences, Bristol Medical School, University of Bristol, Bristol, UK; 6 Research Council, Swiss National Science Foundation, Bern, Switzerland

**Keywords:** statistics & research methods, health policy, health economics

## Abstract

**Objectives:**

To trial a simplified, time and cost-saving method for remote evaluation of fellowship applications and compare this with existing panel review processes by analysing concordance between funding decisions, and the use of a lottery-based decision method for proposals of similar quality.

**Design:**

The study involved 134 junior fellowship proposals for postdoctoral research (‘Postdoc.Mobility’). The official method used two panel reviewers who independently scored the application, followed by triage and discussion of selected applications in a panel. Very competitive/uncompetitive proposals were directly funded/rejected without discussion. The simplified procedure used the scores of the two panel members, with or without the score of an additional, third expert. Both methods could further use a lottery to decide on applications of similar quality close to the funding threshold. The same funding rate was applied, and the agreement between the two methods analysed.

**Setting:**

Swiss National Science Foundation (SNSF).

**Participants:**

Postdoc.Mobility panel reviewers and additional expert reviewers.

**Primary outcome measure:**

Per cent agreement between the simplified and official evaluation method with 95% CIs.

**Results:**

The simplified procedure based on three reviews agreed in 80.6% (95% CI: 73.9% to 87.3%) of applicants with the official funding outcome. The agreement was 86.6% (95% CI: 80.6% to 91.8%) when using the two reviews of the panel members. The agreement between the two methods was lower for the group of applications discussed in the panel (64.2% and 73.1%, respectively), and higher for directly funded/rejected applications (range: 96.7%–100%). The lottery was used in 8 (6.0%) of 134 applications (official method), 19 (14.2%) applications (simplified, three reviewers) and 23 (17.2%) applications (simplified, two reviewers). With the simplified procedure, evaluation costs could have been halved and 31 hours of meeting time saved for the two 2019 calls.

**Conclusion:**

Agreement between the two methods was high. The simplified procedure could represent a viable evaluation method for the Postdoc.Mobility early career instrument at the SNSF.

**Strengths and limitations of this study**

- The study examined the outcome between a simplified and the official evaluation procedure for junior fellowship applications for different research disciplines.
- The study discussed the agreement between the two evaluation methods in the context of the general uncertainty around peer review and estimated the costs and time that could have been saved with the simplified evaluation procedure.
- It is the first study to provide insight into lottery-based decisions in the context of the evaluation of junior fellowship applications.
- The study lacks statistical power because the number of applications was relatively small.
- The study addressed the specific context and evaluation of the Swiss National Science Foundation Postdoc.Mobility funding scheme, results may thus not be generalisable to other funding programmes.

## Introduction

Peer review of grant proposals is costly and time-consuming. The burden on the scientific system is increasing, affecting funders, reviewers and applicants.^1 2^ In response, researchers have studied the review process and examined simplifications. For example, Snell^3^ studied the number of reviewers and consistency of decisions and found that five evaluators represented an optimal tradeoff. Graves *et al*^4^ assessed the reliability of decisions made by evaluation panels of different sizes. They concluded that reliability was greatest with about 10 panel members. Herbert *et al*^5^ compared smaller panels and shorter research proposals with the standard review procedure. The agreement was about 75% between simplified and standard procedures. As an alternative to face-to-face (FTF) panels, the use of virtual, online meetings has also been examined. Bohannon^6^ reported that at the National Science Foundation and National Institutes of Health (NIH), virtual meetings could reduce costs by one-third. Gallo *et al*^7^ compared teleconferencing with FTF meetings and found only few differences in the scoring of the applications. Later studies also found that virtual and FTF panels produce comparable outcomes.^8–10^

With virtual formats, panel members still need to attend time-consuming meetings. Using the reviewers’ written assessments without FTF or virtual panel discussions would simplify the process further. Fogelholm *et al*^11^ reported that results were similar when using panel consensus or the mean of reviewer scores. Obrecht *et al*^12^ noted that panel review changed the funding outcome of only 11% of applications. Similarly, Carpenter *et al*^8^ found that the impact of discussions was small, affecting the funding outcome of about 10% of applications. Pina *et al*^13^ studied Marie Curie Actions applications and concluded that ranking applications based on reviewer scores might work for some but not all disciplines. In the Humanities, Social and Economic Sciences, an exchange between reviewers may be particularly relevant. The triaging of applications has also been examined: after an initial screening, non-competitive and very competitive proposals are either directly rejected or funded. Vener *et al*^14^ validated the triage model of the NIH and found that the likelihood of erroneously discarding a competitive proposal was very small. Bornmann *et al*’s^15^ findings on a multistage fellowship selection process also supported the use of a triage.

Mandated by the government, the Swiss National Science Foundation (SNSF) is Switzerland’s foremost funding agency, supporting scientific research in all disciplines. Following changes to the career-funding portfolio, the SNSF will experience a significant increase of applications for the junior ‘Postdoc.Mobility’ fellowship scheme, which offers postdoctoral researchers a stay at a research institution abroad for up to 24 months. The scheme enables junior postdocs to deepen their scientific knowledge and increase their scientific independence during a research stay abroad. The aim of this work was to compare the evaluation of applications by expert review, triage, and discussion in an evaluation panel with expert reviews only.

## Methods

### Sample

We included applications submitted for the August 2019 Postdoc.Mobility fellowship call. We also included applications by Postdoc.Mobility fellows for a return grant to facilitate their return to Switzerland. Both fellowship and return grants were evaluated according to the same criteria by one of five panels: Humanities, Social Sciences, STEM (Science, Technology, Engineering and Mathematics), Biology or Medicine.

### Study design

**Figure 1:**
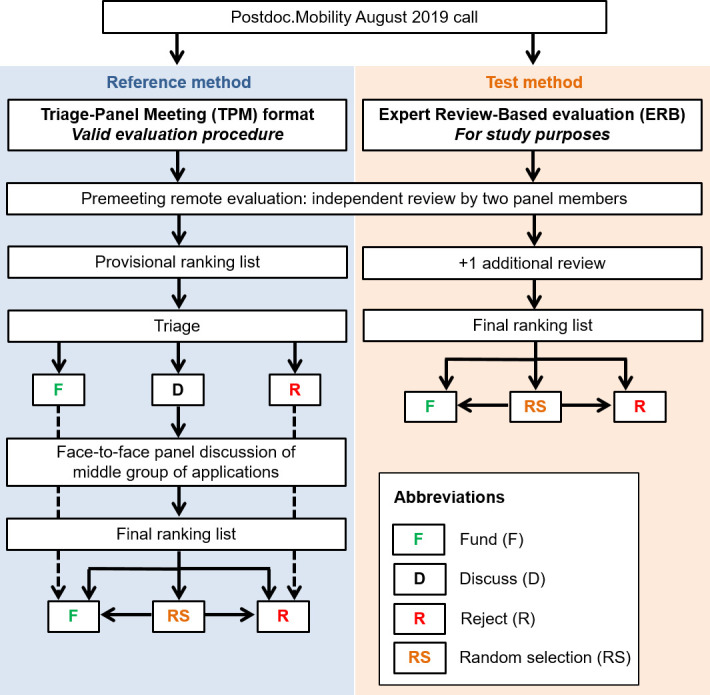
Design of the study comparing the ERB evaluation with the TPM format. The ERB and the TPM were dependent in terms of the two assigned panel reviewers per application. The third reviewers were only added for the ERB, their assessments were not considered for the TPM and therefore the official funding outcome. ERB, expert review based; TPM, triage-panel meeting.

We compared funding outcomes based on the official, legally binding evaluation with a simulated, hypothetical evaluation. The official evaluation was based on the triage of applications based on expert reviews, followed by a discussion of the meritorious applications in an FTF panel: the triage-panel meeting (TPM) format (figure 1). In a first step, each proposal was independently reviewed and scored by two panel members. For the assessment, the evaluation criteria defined in the Postdoc.Mobility regulations^16^ were applied. The criteria address different aspects of the applicant, the proposed research project and the designated research location. Panel members used a 6-point scale: outstanding=6 points, excellent=5 points, very good=4 points, good=3 points, mediocre=2 points and poor=1 point. Applications were then allocated to three groups based on the ranking of the mean scores given to each proposal: Fund without further discussion (F in figure 1), Discuss in panel meeting (D) and Reject (R). Panel members could request that applications in the F or R group are reallocated to D and discussed. In a second step, the D proposals were discussed in the FTF panel meeting, ranked and funded or rejected. Random selection (RS in figure 1) could be used to fund or reject proposals of similar quality close to the funding threshold if the panel could not reach a decision. Funding decisions were based on the standard two-stage method, which included FTF panel meetings (TPM).

The simulated alternative procedure consisted only of the first step, that is, was entirely based on the ranking of proposals based on the expert reviews: the expert review-based (ERB) evaluation. In addition to the two panel members, a third expert reviewer who was not a member of the panel assessed the proposal. The same 6-point scale was used. The proposals were then allocated to one of three groups based on the mean scores (F, RS and R in figure 1). Random selection was used whenever the funding line went through a group of two or more applications with identical scores. The funding rate of the TPM was applied to the simulated ERB method.

### Data analysis

To determine the agreement between the two evaluation methods, we used 2×2 contingency tables. We calculated the simple agreement with 95% CIs, which were generated using a bootstrap algorithm.^5^ We also examined the agreement between the TPM and the ERB approach using only the assessments from the two panel members, thus excluding the assessment from the third reviewer. We calculated discipline-specific and gender-specific levels of agreement. We used χ^2^ tests for categorical data to test whether the agreement differed between these mutually exclusive groups.

### Costs

We determined the costs related to the evaluation. The costs comprised expenses related to the scientific assessment of the individual applications and the panel meetings. The SNSF compensates panel reviewers with US$275 per scientific assessment. Panel reviewers further receive a meeting allowance of up to US$550 depending on the duration of the meeting. Further, the SNSF reimburses travel expenses and accommodation costs. The five panels included 96 members and met twice in 2019.

### Patient and public involvement

This analysis was based on expert review reports submitted to the SNSF. No patients were involved in developing the research question, outcome measures and overall design of the study.

## Results

### Study sample and success rates

The sample consisted of 134 applications, including 124 fellowship applications and 10 requests for a return grant. The mean age of applicants was 32.7 years (SD 3.2 years) in men and 33.5 years (SD 2.8 years) in women. Each reviewer received a mean of 2.5 (SD 1.4) applications to evaluate.

Table 1 shows the summary statistics of applications and success rates across disciplines, genders and the three evaluation methods: the legally binding TPM format and the simulated ERB evaluations with three or two reviewers. Most applications came from biology, followed by the STEM disciplines and the Social Sciences. Almost two-thirds of applications came from men. With TPM, success rates were slightly higher in women (60.4%) than in men (50.0%). This was driven by the middle group of applications that were discussed in the panels, where the success rate of women overall was 66.7% (24 of 67 applicants were women in this group). Success rates were similar across disciplines, ranging from 56.2% in the Humanities to 52.2% in the Social Sciences. By design, overall success rates were the same with the ERB evaluations; however, the difference between genders was smaller with ERB than with TPM (table 1).

**Table 1:** Success rates by gender of applicants, by discipline and type of evaluation

	All applicants		Women		Men	
Discipline	N	N funded (%)	N	N funded (%)	N	N funded (%)
TPM						
All disciplines	134	72 (53.7)	48	29 (60.4)	86	43 (50.0)
Humanities	16	9 (56.2)	9	4 (44.4)	7	5 (71.4)
Social Sciences	23	12 (52.2)	10	7 (70.0)	13	5 (38.5)
STEM	35	19 (54.3)	10	6 (60.0)	25	13 (52.0)
Biology	40	21 (52.5)	14	8 (57.1)	26	13 (50.0)
Medicine	20	11 (55.0)	5	4 (80.0)	15	7 (46.7)
ERB (three reviewers)*						
All disciplines	134	72 (53.7)	48	27 (56.3)	86	45 (52.3)
Humanities	16	9 (56.3)	9	5 (55.6)	7	4 (57.1)
Social Sciences	23	12 (52.2)	10	6 (60.0)	13	6 (46.2)
STEM	35	19 (54.3)	10	4 (40.0)	25	15 (60.0)
Biology	40	21 (52.5)	14	8 (57.1)	26	13 (50.0)
Medicine	20	11 (55.0)	5	4 (80.0)	15	7 (46.7)
ERB (two reviewers)†						
All disciplines	134	72 (53.7)	48	25 (52.1)	86	47 (54.7)
Humanities	16	9 (56.3)	9	5 (55.6)	7	4 (57.1)
Social Sciences	23	12 (52.2)	10	6 (60.0)	13	6 (46.2)
STEM	35	19 (54.3)	10	4 (40.0)	25	15 (60.0)
Biology	40	21 (52.5)	14	7 (50.0)	26	14 (53.8)
Medicine	20	11 (55.0)	5	3 (60.0)	15	8 (53.3)

*Two of the three expert reviewers were also members of the evaluation panel.

†Both expert reviewers were also members of the evaluation panel.

ERB, expert review based; N, number of applications; STEM, Science, Technology, Engineering, Mathematics; TPM, triage-panel meeting.

### Agreement between evaluation by ERB or TPM

Comparing the ERB evaluation based on three reviewers with the standard TPM format, the agreement overall was 80.6% (95% CI: 73.9% to 87.3%). The agreement was highest in the Medicine panel (90.0%; 95% CI: 75% to 100%) and lowest in the Social Sciences panel (73.9%; 95% CI: 56.5% to 91.3%). However, the statistical evidence for differences in agreement between panels was weak (p=0.58, table 2). As expected, the agreement was higher when comparing the ERB evaluation based on the two panel members with TPM. Overall, for two reviews, the agreement was 86.6% (95% CI: 80.6% to 91.8%). It ranged from 75.0% (95% CI: 50.0% to 93.8%) in the Humanities panel to 91.3% (95% CI: 78.3% to 100%) in the Social Sciences panel. Again, there was no evidence for differences in agreement between panels (p=0.51). Both for ERB evaluation with three and two reviewers, the agreement was slightly higher for women than for men (p>0.70, table 3).

**Table 2:** Agreement between the simulated ERB evaluation and the TPM format, by discipline

Discipline	N	Funded by TPM		Agreement (%) (95% CI)
Funded by ERB (three reviewers)*		Yes	No	
All disciplines	Yes	59	13	80.6
	No	13	49	(73.9 to 87.3)
Humanities	Yes	7	2	75.0
	No	2	5	(50.0 to 93.8)
Social Sciences	Yes	9	3	73.9
	No	3	8	(56.5 to 91.3)
STEM	Yes	15	4	77.1
	No	4	12	(62.9 to 91.4)
Biology	Yes	18	3	85.0
	No	3	16	(72.5 to 95)
Medicine	Yes	10	1	90.0
	No	1	8	(75 to 100)
P value				0.58
Funded by ERB (two reviewers)†				
All disciplines	Yes	63	9	86.6
	No	9	53	(80.6 to 91.8)
Humanities	Yes	7	2	75.0
	No	2	5	(50.0 to 93.8)
Social Sciences	Yes	11	1	91.3
	No	1	10	(78.3 to 100)
STEM	Yes	16	3	82.9
	No	3	13	(68.6 to 94.3)
Biology	Yes	19	2	90.0
	No	2	17	(80 to 97.5)
Medicine	Yes	10	1	90.0
	No	1	8	(75 to 100)
P value				0.51

P values for differences in agreement across disciplines from χ^2^ test.

*Two of the three expert reviewers were also members of the evaluation panel.

†Both expert reviewers were also members of the evaluation panel.

ERB, expert review based; N, number of applications; STEM, Science, Technology, Engineering, Mathematics; TPM, triage-panel meeting.

**Table 3:** Agreement between the simulated ERB evaluation and the TPM format, by gender

Gender		Funded by TPM		Agreement (%) (95% CI)
Funded by ERB (three reviewers)*		Yes	No	
Women	Yes	24	3	83.3
	No	5	16	(72.9 to 93.8)
Men	Yes	35	10	79.1
	No	8	33	(69.8 to 87.2)
P value				0.71
Funded by ERB (two reviewers)†				
Women	Yes	24	1	87.5
	No	5	18	(77.1 to 95.8)
Men	Yes	39	8	86.0
	No	4	35	(77.9 to 93.0)
P value				0.99

P values for differences in agreement across genders from χ^2^ test.

*Two of the three expert reviewers were also members of the evaluation panel.

†Both expert reviewers were also members of the evaluation panel.

ERB, expert review based; N, number of applications; STEM, Science, Technology, Engineering, Mathematics; TPM, triage-panel meeting.

In table 4, we calculated agreement separately for the triage categories: Fund (F), Discuss (D) and Reject (R). With the ERB evaluation based on three reviewers, agreements for F and R were close to 100% (97.3% and 96.7%, respectively) but considerably lower for D: 64.2% (95% CI: 52.2% to 76.1%), with p<0.001 for differences in agreement across categories. For ERB evaluation with two reviewers (the two panel members), the agreement was 100% for F and R, but 73.1% (95% CI: 62.7% to 83.6%) for D, with p<0.001 for differences in agreement.

**Table 4:** Agreement between the simulated ERB evaluation and the TPM format, by triage results

Triage result		Funded by TPM		Agreement (%) (95% CI)
Funded by ERB (three reviewers)*		Yes	No	
Fund (F)	Yes	36	0	97.3
	No	1	0	(91.9 to 100)
Discuss (D)	Yes	23	12	64.2
	No	12	20	(52.2 to 76.1)
Reject (R)	Yes	0	1	96.7
	No	0	29	(90.0 to 100)
P value				<0.001
Funded by ERB (two reviewers)†				
Fund (F)	Yes	37	0	100
	No	0	0	
Discuss (D)	Yes	26	9	73.1
	No	9	23	(62.7 to 83.6)
Reject (R)	Yes	0	0	100
	No	0	30	
P value				<0.001

P values for differences in agreement across triage groups from χ^2^ test.

*Two of the three expert reviewers were also members of the evaluation panel.

†Both expert reviewers were also members of the evaluation panel.

ERB, expert review based; N, number of applications; STEM, Science, Technology, Engineering, Mathematics; TPM, triage-panel meeting.

### Random selection in TPM and ERB evaluation

With the standard TPM evaluation, only 8 (11.9%) of the 67 applicants in the D group, or 8 (6.0%) of 134 applicants were entered into a lottery of whom 4 were funded. With the simulated ERB evaluation based on three reviewers, 19 (14.2%) of the 134 applicants would have entered the lottery, and with the ERB with two reviewers 23 (17.2%) applications would have been subjected to random selection.

### Cost and time savings

We determined the resources that could be saved with the use of an ERB evaluation compared with the TPM. By comparison with the current valid TPM evaluation procedure for the Postdoc.Mobility, we calculated that about US$91 000 related to the holding of meetings could have been saved if an ERB evaluation had been used for the two Postdoc.Mobility calls in 2019. This saving corresponds to 55% of total costs. Moreover, the holding of all panel sessions in 2019 amounted to 31 meeting hours, a significant workload that could have been avoided using the ERB approach. Lastly, the funding decisions could have been communicated at least 1 month earlier with ERB, reducing the time to notification by about 20% compared with TPM.

## Discussion

In this comparative study of the evaluation of early-career funding applications, we found that the simulated funding outcomes of a simplified, ERB approach agreed well with the official funding outcomes based on the standard, time-tested TPM format. Applications for fellowships covered a wide range of disciplines, from the Humanities and Social Sciences to STEM, Biology and Medicine. The agreement was very high for proposals which, in the TPM evaluation, were either allocated to the fund or reject categories, but lower in the middle category of proposals that were discussed by the panels. More applicants entered the lottery with the simplified ERB approach than with TPM evaluation. Finally, the simplified ERB evaluation approach was associated with a substantial reduction in costs. Overall, our results support the notion that a sound evaluation of early-career funding applications is possible with an ERB approach.

Although panel review is considered as a ‘de facto’ standard, the consistency of decisions from panels has been shown to be limited. For example, previous work by Cole *et al*,^17^ Hodgson,^18^ Fogelholm *et al*^11^ and Clarke *et al*^19^ found an agreement of 65% to 83% between two independent panels evaluating the same set of applications. Thus, in these studies, the funding outcome also depended on the panel that evaluated the application, and not only on the scientific content. Against this background, the agreement of over 80% between ERB and TPM in this study is remarkable. Among the different discipline-specific review panels, our results showed a slightly lower agreement in the Humanities and Social Sciences compared with Life Sciences and Medicine. These differences did not reach conventional levels of statistical significance but were in line with previous findings reported by Pina *et al.*^13^

In the middle group of applications based on the triage step of TPM, the agreement was lower; 64% with three reviewers and 73% with the two reviewers. This is not surprising considering the results from previous studies that suggest that peer review has difficulties in discriminating between applications that are neither clearly excellent nor clearly non-competitive.^20–22^ Agreement between ERB and TPM was also generally lower with ERB using three reviewers than with ERB with two reviewers. An additional reviewer may introduce a different viewpoint. Also, the third reviewer was not a member of the corresponding panel, and not involved in previous panel discussions, which have led to some degree of calibration between assessments of panel members. Such calibration is more difficult to achieve with a remote, ERB approach. However, information and briefing sessions could be held to compensate for the lack of FTF panel meetings. Of note, previous studies reported that reviewers appreciated the social aspects and the camaraderie in FTF settings and that physical meetings are important for building trust among the evaluators.^8 9^

We found that the panel discussions in the TPM format resulted in higher success rates for women compared with the ERB format. Gender equality is a key concern at the SNSF, which is committed to promoting women in research. The panels will have been aware of the under-representation of female researchers in certain areas, for example, the STEM disciplines, and the SNSF’s agenda to promote women. It is, therefore, possible that during the panel deliberations and for funding decisions, the gender of applicants was taken into account in addition to the quality of the proposal.

We estimated that about US$91 000 could have been saved for the two Postdoc.Mobility calls in 2019 if they had been evaluated by ERB rather than by TPM. The meeting costs represented about 55% of the total evaluation costs. In other words, the ERB evaluation based on the two panel reviewers would have cut the expenses by more than half. The experience described here with the junior Postdoc.Mobility fellowship scheme indicates that substantial cost savings could also result from simplifications in the evaluation of other funding instruments at the SNSF. Also, ERB would have reduced the time to communication of funding decisions, and unsuccessful applicants could thus have planned their next steps earlier. However, any such changes need to be considered carefully. The quality of the evaluation should not be allowed to be compromised because costs may be reduced.

To the best of our knowledge, the Health Research Council of New Zealand (HRC-NZ),^23^ the Volkswagen Foundation^24^ and recently the Austrian Research Fund FWF^25^ are the only funders that have used or examined the use of a random selection element in the evaluation process of funding instruments, with a focus on transformative research or unconventional research ideas. The random selection for decisions on applications close to the funding threshold could avoid bias if evaluation criteria do not allow any further differentiation for a small set of similarly qualified applications.^22 26^ The applicants were informed about the possible random selection and the evaluation process thus complied with the San Francisco Declaration on Research Assessment,^27^ which states that funders must be explicit about assessment criteria. In this context, evaluation criteria could be weighted, or additional strategic criteria be used in the selection process if defined a priori and communicated to applicants. However, weighting or additional criteria could also lead to tied applications and thus require a lottery decision. There was some reservation on the random selection approach among some panel members, but acceptance grew over time. Of note, panels applied the random selection only in a few cases, in 8 (6.0%) of 134 applications. In the context of the Explorer Grant scheme of the HRC-NZ, Liu *et al*^28^ recently reported that most applicants agreed with the use of a random selection. In this study, the SNSF received a few unsolicited questions about the procedure but otherwise no negative or positive reactions to the use of random selection were received from applicants.

Our study has several limitations. It addressed the specific context of the SNSF Postdoc.Mobility funding scheme and results may not be generalisable to other funding instruments. The sample size was relatively small, and the study lacked statistical power, for example, to examine differences in agreement between TPM and ERB evaluation across disciplines. The two evaluation methods were not independent, since the two assessments of the panel reviewers were used for both methods. We were relying on reviewer evaluation scores which might not always perfectly reflect the quality of the proposed project, might be biased and depend on the reviewers’ previous experience with grant evaluation. However, our study design allowed us to investigate the impact of panel meetings on funding outcomes compared with an ERB approach. This study provides further insights into peer review and a modified lottery approach selection in the context of the evaluation of fellowship applications. More research on the limitations inherent in peer review and grant evaluation is urgently needed. Funders should be creative when investigating the merit of different evaluation strategies.^29^

## Conclusions

In conclusion, we simulated an ERB approach in the evaluation of the junior Postdoc.Mobility funding scheme at the SNSF and compared the funding outcome to the standard TPM format, which has been in use for many years. We found an overall high agreement between the two methods. Discrepancies were mainly observed in the middle group of applications that were discussed in the panel meetings. Based on the evidence that peer review has difficulties in making fine-grained differentiations between meritorious applications,^20–22^ we are unsure which method performs better. Our findings indicate that the ERB approach represents a viable evaluation method for the Postdoc.Mobility selection process that could save cost and time which could be invested in science and research.

## Supplementary Material

Reviewer comments

Author's manuscript

## Data Availability

Data are available in a public, open access repository. An anonymised data set can be found on Zenodo (https://doi.org/10.5281/zenodo.5546361).
