# Supplementary material for: Face-to-face panel meetings versus remote evaluation of fellowship applications: simulation study at the Swiss National Science Foundation
Source: BMJ Open. 2021 May 5;11(5):e047386. doi: 10.1136/bmjopen-2020-047386 (PMC8103360; doi:10.1136/bmjopen-2020-047386)
Supplement: Author's manuscript [file bmjopen-2020-047386.draft_revisions.pdf]

BMJ Open is committed to open peer review. As part of this commitment we make the peer review history of every article we publish publicly available.

When an article is published we post the peer reviewers' comments and the authors' responses online. We also post the versions of the paper that were used during peer review. These are the versions that the peer review comments apply to.

The versions of the paper that follow are the versions that were submitted during the peer review process. They are not the versions of record or the final published versions. They should not be cited or distributed as the published version of this manuscript.

BMJ Open is an open access journal and the full, final, typeset and author-corrected version of record of the manuscript is available on our site with no access controls, subscription charges or pay-per-view fees (http://bmjopen.bmj.com).

If you have any questions on BMJ Open's open peer review process please email info.bmjopen@bmj.com

BMJ Open

How to best evaluate applications for junior fellowships? Remote evaluation and face-to-face panel meetings compared

Journal:	*BMJ Open*
Manuscript ID	bmjopen-2020-047386
Article Type:	Original research
Date Submitted by the Author:	30-Nov-2020
Complete List of Authors:	Bieri, Marco; Swiss National Science Foundation,<br>Roser, Katharina; University of Lucerne,<br>Heyard, Rachel; Swiss National Science Foundation<br>Egger, Matthias; University of Bern, Institute of Social & Preventive Medicine
Keywords:	STATISTICS & RESEARCH METHODS, Health policy < HEALTH SERVICES ADMINISTRATION & MANAGEMENT, HEALTH ECONOMICS

SCHOLARONE™
Manuscripts

I, the Submitting Author has the right to grant and does grant on behalf of all authors of the Work (as defined in the below author licence), an exclusive licence and/or a non-exclusive licence for contributions from authors who are: i) UK Crown employees; ii) where BMJ has agreed a CC-BY licence shall apply, and/or iii) in accordance with the terms applicable for US Federal Government officers or employees acting as part of their official duties; on a worldwide, perpetual, irrevocable, royalty-free basis to BMJ Publishing Group Ltd ("BMJ") its licensees and where the relevant Journal is co-owned by BMJ to the co-owners of the Journal, to publish the Work in this journal and any other BMJ products and to exploit all rights, as set out in our licence.

The Submitting Author accepts and understands that any supply made under these terms is made by BMJ to the Submitting Author unless you are acting as an employee on behalf of your employer or a postgraduate student of an affiliated institution which is paying any applicable article publishing charge ("APC") for Open Access articles. Where the Submitting Author wishes to make the Work available on an Open Access basis (and intends to pay the relevant APC), the terms of reuse of such Open Access shall be governed by a Creative Commons licence – details of these licences and which Creative Commons licence will apply to this Work are set out in our licence referred to above.

Other than as permitted in any relevant BMJ Author's Self Archiving Policies, I confirm this Work has not been accepted for publication elsewhere, is not being considered for publication elsewhere and does not duplicate material already published. I confirm all authors consent to publication of this Work and authorise the granting of this licence.

For peer review only

Original Manuscript

How to best evaluate applications for junior fellowships? Remote evaluation and face-to-face panel meetings compared

Marco Bieri[1¶*], Katharina Roser[1,2¶], Rachel Heyard[1], Matthias Egger[1,3,4]

[1]Swiss National Science Foundation, Berne, Switzerland

[2]Department of Health Sciences and Medicine, University of Lucerne, Lucerne, Switzerland

[3]Institute of Social and Preventive Medicine, University of Berne, Berne, Switzerland

[4]Population Health Sciences, Bristol Medical School, University of Bristol, Bristol, UK

¶These authors contributed equally to this work

*Corresponding author:

Marco Bieri

Swiss National Science Foundation

Wildhainweg 3

CH-3001 Berne

marco.bieri@snf.ch

Word count: abstract 291 words, box on strengths and limitations 119 words, main text 2835 words, 29 references, 4 tables, 1 figure.

For peer review only

Abstract

Objectives

To test a simplified evaluation of fellowship proposals by analyzing the agreement of funding decisions with the official evaluation, and to examine the use of a lottery-based decision for proposals of similar quality.

Design

The study involved 134 junior fellowship proposals (Postdoc.Mobility). The official method used two panel reviewers who independently scored the application, followed by triage and discussion of selected applications in a panel. Very competitive/uncompetitive proposals were directly funded/rejected without discussion. The simplified procedure used the scores of the two panel members, with or without the score of an additional, third expert. Both methods could further use a lottery to decide on applications of similar quality close to the funding threshold. The same funding rate was applied, and the agreement between the two methods analyzed.

Setting

Swiss National Science Foundation (SNSF).

Participants

Postdoc.Mobility panel reviewers and additional expert reviewers.

Primary outcome measure

Per cent agreement between the simplified and official evaluation method with 95% confidence intervals (95% CI).

Results

The simplified procedure based on three reviews agreed in 80.6% (95% CI 73.9-87.3) with the official funding outcome. The agreement was 86.6% (95% CI 80.8-92.4) when using the two reviews of the panel members. The agreement between the two methods was lower for the group of applications discussed in the panel (64.2% and 73.1%, respectively), and higher

For peer review only - http://bmjopen.bmj.com/site/about/guidelines.xhtml
For peer review only

for directly funded/rejected applications (range 96.7% to 100%). The lottery was used in eight (6.0%) of 134 applications (official method), 19 (14.2%) applications (simplified, three reviewers) and 23 (17.2%) applications (simplified, two reviewers). With the simplified procedure, evaluation costs could have been halved and 31 hours of meeting time saved for the two 2019 calls.

Conclusion

Agreement between the two methods was high. The simplified procedure could represent a viable evaluation method for the Postdoc.Mobility early career instrument at the SNSF.

For peer review only

Strengths and limitations of this study

- The study examined the outcome between a simplified and the official evaluation procedure for junior fellowship applications for different research disciplines.
- The study discussed the agreement between the two evaluation methods in the context of the general uncertainty around peer review and estimated the costs that could have been saved with the simplified evaluation procedure.
- It is the first study to provide insight into lottery-based decisions in the context of the evaluation of junior fellowship applications.
- The study lacks statistical power because the sample size of applications was relatively small.
- The study addressed the specific context and evaluation of the SNSF Postdoc.Mobility funding scheme, results may thus not be generalizable to other funding programs.

For peer review only - http://bmjopen.bmj.com/site/about/guidelines.xhtml

Introduction

Peer review of grant proposals is costly and time-consuming. The burden on the scientific system is increasing, affecting funders, reviewers, and applicants [1,2]. In response, researchers have studied the review process and examined simplifications. For example, Snell [3] studied the number of reviewers and consistency of decisions and found that five evaluators represented an optimal tradeoff. Graves et al. [4] assessed the reliability of decisions made by evaluation panels of different sizes. They concluded that reliability was greatest with about ten panel members. Herbert et al. [5] compared smaller panels and shorter research proposals with the standard review procedure. The agreement was about 75% between simplified and standard procedures. As an alternative to face-to-face (FTF) panels, the use of virtual, online meetings has also been examined. Bohannon [6] reported that at the National Science Foundation (NSF) and National Institutes of Health (NIH), virtual meetings could reduce costs by one-third. Gallo et al. [7] compared teleconferencing with FTF meetings and found only few differences in the scoring of the applications. Later studies also found that virtual and FTF panels produce comparable outcomes [8–10].

With virtual formats, panel members still need to attend time-consuming meetings. Using the reviewers' written assessments without FTF or virtual panel discussions would simplify the process further. Fogelholm et al. [11] reported that results were similar when using panel consensus or the mean of reviewer scores. Obrecht et al. [12] noted that panel review changed the funding outcome of only 11% of applications. Similarly, Carpenter et al. [8] found that the impact of discussions was small, affecting the funding outcome of about 10% of applications. Pina et al. [13] studied Marie Curie Actions applications and concluded that ranking applications based on reviewer scores might work for some but not all disciplines. In the humanities, social and economic sciences, an exchange between reviewers may be particularly relevant. The triaging of applications has also been examined: after an initial screening, noncompetitive and very competitive proposals are either directly rejected or funded. Vener et al. [14] validated the triage model of the NIH and found that the likelihood of erroneously discarding a competitive proposal was very small. Bornmann et al.'s [15] findings on a multi-stage fellowship selection process also supported the use of a triage.

Mandated by the government, the Swiss National Science Foundation (SNSF) is Switzerland's foremost funding agency, supporting scientific research in all disciplines. Following innovations in career funding, the SNSF will experience a significant increase of applications for the junior "Postdoc.Mobility" fellowship scheme, which offers postdoctoral

For peer review only - http://bmjopen.bmj.com/site/about/guidelines.xhtml

researchers a stay at a research institution abroad for up to 24 months. The aim of this work was to compare the evaluation of applications by expert review, triage, and discussion in an evaluation panel with expert reviews only.

Methods

Sample

We included applications submitted for the August 2019 Postdoc.Mobility fellowship call. We also included applications by Postdoc.Mobility fellows for a return grant to facilitate their return to Switzerland. Both, fellowship and return grants were evaluated according to the same criteria by the Humanities panel, the Social Sciences panel, Science, Technology, Engineering, Mathematics (STEM) panel, the Biology or Medicine panels.

Study design

We compared funding outcomes based on the official, legally binding evaluation with a simulated, hypothetical evaluation. The official evaluation was based on the triage of applications based on expert reviews, followed by a discussion of the meritorious applications in an FTF panel: the Triage-Panel Meeting (TPM) format (Figure 1). In a first step, each proposal was independently reviewed and scored by two panel members. For the assessment, the evaluation criteria defined in the Postdoc.Mobility regulations [16] were applied. The criteria address different aspects of the applicant, the proposed research project, and the designated research location. Panel members used a 6-point scale: outstanding=6 points, excellent=5 points, very good=4 points, good=3 points, mediocre=2 points, poor=1 point. Applications were then allocated to three groups based on the ranking of the mean scores given to each proposal: Fund without further discussion (F in Figure 1), Discuss in panel meeting (D), and Reject (R). Panel members could request that applications in the F or R group are reallocated to D and discussed. In a second step, the D proposals were discussed in the FTF panel meeting, ranked and funded or rejected. Random Selection (RS in Figure 1) could be used to fund or reject proposals of similar quality close to the funding threshold if the panel could not reach a decision. Funding decisions were based on the standard two-stage method, which included FTF panel meetings (TPM).

The simulated alternative procedure consisted only of the first step, i.e., was entirely based on the ranking of proposals based on the expert reviews: the Expert Review-Based (ERB) evaluation. In addition to the two panel members, a third expert reviewer who was not

For peer review only - http://bmjopen.bmj.com/site/about/guidelines.xhtml

a member of the panel assessed the proposal. The same 6-point scale was used. The proposals were then allocated to one of three groups based on the mean scores (F, RS, and R in Figure 1). Random selection was used whenever the funding line went through a group of two or more applications with identical scores. The funding rate of the TPM was applied to the simulated ERB method.

Data analysis

To determine the agreement between the two evaluation methods, we used 2x2 contingency tables. We calculated the simple agreement with 95% Wald confidence intervals (CI) for proportions. We also examined the agreement between the TPM and the ERB approach using only the assessments from the two panel members, thus excluding the assessment from the third reviewer. We calculated discipline- and gender-specific levels of agreement and tested for differences in agreement between disciplines and gender using chi-squared tests for categorical data.

Costs

We determined the costs related to the evaluation. The costs comprised expenses related to the scientific assessment of the individual applications and the panel meetings. The SNSF compensates panel reviewers with USD 275 per scientific assessment. Panel reviewers further receive a meeting allowance of up to USD 550 depending on the duration of the meeting. Further, the SNSF reimburses travel expenses and accommodation costs. The five panels included 96 members and met twice in 2019.

Ethics approval

The Ethics Committee of the Canton of Berne confirmed that the study does not fall under the Federal Act on Research involving Human Beings. No reviewer, applicant or application can be identified from this study.

Patient and Public Involvement

This analysis was based on expert review reports submitted to the Swiss National Science Foundation. No patients were involved in developing the research question, outcome measures and overall design of the study.

For peer review only - http://bmjopen.bmj.com/site/about/guidelines.xhtml

Results

Study sample and success rates

The sample consisted of 134 applications, including 124 fellowship applications and ten requests for a return grant. The mean age of applicants was 32.7 years (SD 3.2 years) in men and 33.5 years (SD 2.8 years) in women. Each reviewer received a mean of 2.5 (SD 1.4) applications to evaluate.

Table 1 shows the distribution of applications and success rates across disciplines, genders and the three evaluation methods: the legally binding TPM format and the simulated ERB evaluations with three or two reviewers. Most applications came from biology, followed by the STEM disciplines and the social sciences. Almost two-thirds of applications came from men. With TPM, success rates were slightly higher in women (60.4%) than in men (50.0%). This was driven by the middle group of applications that were discussed in the panels, where the success rates of women overall was 66.7% (24 of 67 applicants were women in this group). Success rates were similar across disciplines, ranging from 56.2% in the humanities to 52.2% in the social sciences. By design, overall success rates were the same with the ERB evaluations; however, the difference between genders was smaller with ERB than with TPM (Table 1).

Agreement between evaluation by ERB or TPM

Comparing the ERB evaluation based on three reviewers with the standard TPM format, the agreement overall was 80.6% (95% CI 73.9-87.3). The agreement was highest in the Medicine panel (90.0%; CI 76.9-100), and lowest in the Social Sciences panel (73.9%; CI 56.0-91.8). However, the statistical evidence for differences in agreement between panels was weak (P=0.58, Table 2). As expected, the agreement was higher when comparing the ERB evaluation based on the two panel members with TPM. Overall, for two reviews, the agreement was 86.6% (95% CI 80.8-92.4). It ranged from 75.0% (CI 53.8-96.2) in the Humanities panel to 91.3% (CI 79.8-100) in the Social Sciences panel (P=0.51). Both for ERB evaluation with three and two reviewers, the agreement was slightly higher for women than for men (P>0.70, Table 3).

In Table 4, we calculated agreement separately for the triage categories: Fund (F), Discuss (D), Reject (R). With the ERB evaluation based on three reviewers, agreements for F and R were close to 100% (97.3% and 96.7%, respectively) but considerably lower for D:

For peer review only - http://bmjopen.bmj.com/site/about/guidelines.xhtml

64.2% (95% CI 52.7-75.7), with P<0.001 for differences in agreement across categories from chi-squared test. For ERB evaluation with two reviewers (the two panel members), the agreement was 100% for F and R, but 73.1% (95% CI 62.5- 83.7) for D, with P<0.001 for differences in agreement.

Random selection in TPM and ERB evaluation

With the standard TPM evaluation, only eight (11.9%) of the 67 applicants in the D group, or eight (6.0%) of 134 applicants were entered into a lottery of whom four were funded. With the simulated ERB evaluation based on three reviewers, 19 (14.2%) of the 134 applicants would have entered the lottery, and with the ERB with two reviewers 23 (17.2%) applications would have been subjected to random selection.

Cost savings

We determined the resources that could be saved with the use of an ERB evaluation compared to the TPM. By comparison with the current valid TPM evaluation procedure for the Postdoc.Mobility, we calculated that about USD 91,000 related to the holding of meetings could have been saved if an ERB evaluation had been used for the two Postdoc.Mobility calls in 2019. This saving corresponds to 55% of total costs. Moreover, the holding of all panel sessions in 2019 amounted to a total duration of 31 hours. This represents a significant workload that could have been eliminated with the use of the ERB approach.

For peer review only

For peer review only - http://bmjopen.bmj.com/site/about/guidelines.xhtml

Discussion

In this comparative study of the evaluation of early-career funding applications, we found that the simulated funding outcomes of a simplified, expert review-based (ERB) approach agreed well with the official funding outcomes based on the standard, time-tested triage and panel meeting (TPM) format. Applications for fellowships covered a wide range of disciplines, from the humanities and social sciences to STEM, biology and medicine. The agreement was very high for proposals which, in the TPM evaluation, were either allocated to the Fund or Reject categories, but lower in the middle category of proposals that were discussed by the panels. More applicants entered the lottery with the simplified ERB approach than with TPM evaluation. Finally, the simplified ERB evaluation approach was associated with a substantial reduction in costs. Overall, our results support the notion that a sound evaluation of early-career funding applications is possible with an ERB approach.

Although panel review is considered as a "de facto" standard, the consistency of decisions from panels has been shown to be limited. For example, previous work by Cole [17], Hodgson [18], Fogelholm [11] and Clarke [19] found an agreement of 65% to 83% between two independent panels evaluating the same set of applications. Thus, in these studies, the funding outcome also depended on the panel that evaluated the application, and not only on the scientific content. Against this background, the agreement of over 80% between ERB and TPM in this study is remarkable. Among the different discipline-specific review panels, our results showed a slightly lower agreement in the humanities and social sciences compared to life sciences and medicine. These differences did not reach conventional levels of statistical significance but were in line with previous findings reported by Pina et al. [13].

In the middle group of applications based on the triage step of TPM, the agreement was lower; 64% with three reviewers and 73% with the two reviewers. This is not surprising considering the results from previous studies that suggest that peer review has difficulties in discriminating between applications that are neither clearly excellent nor clearly noncompetitive [20–22]. Agreement between ERB and TPM was also generally lower with ERB using three reviewers than with ERB with two reviewers. An additional reviewer may introduce a different viewpoint. Also, the third reviewer was not a member of the corresponding panel, and not involved in previous panel discussions, which have led to some degree of calibration between assessments of panel members. Such calibration is more difficult to achieve with a remote, ERB approach. However, information and briefing sessions

For peer review only - http://bmjopen.bmj.com/site/about/guidelines.xhtml

could be held to compensate for the lack of FTF panel meetings. Of note, previous studies reported that reviewers appreciated the social aspects and the camaraderie in FTF settings and that physical meetings are important for building trust among the evaluators [8,9].

We found that the panel discussions in the TPM format resulted in higher success rates for women compared to the ERB format. Gender equality is a key concern at the SNSF, which is committed to promoting women in research. The panels will have been aware of the under-representation of female researchers in certain areas, for example, the STEM disciplines, and the SNSF's agenda to promote women. It is, therefore, possible that during the panel deliberations and for funding decisions, the gender of applicants was taken into account in addition to the quality of the proposal.

We estimated that about USD 91,000 could have been saved for the two Postdoc.Mobility calls in 2019 if they had been evaluated by ERB rather than by TPM. The meeting costs represented about 55% of the total evaluation costs. In other words, the ERB evaluation based on the two panel reviewers would have cut the expenses by more than half. The experience described here with the junior Postdoc.Mobility fellowship scheme indicates that substantial cost savings could also result from simplifications in the evaluation of other funding instruments at the SNSF. However, any such changes need to be considered carefully. The quality of the evaluation should not be allowed to be compromised because costs may be reduced.

To the best of our knowledge, the Health Research Council of New Zealand (HRC-NZ) [23], the Volkswagen Foundation [24], and recently the Austrian Research Fund FWF [25] are the only funders that have used or examined the use of a random selection element in the evaluation process of funding instruments, with a focus on transformative research or unconventional research ideas. The random selection for decisions on applications close to the funding threshold could avoid bias if evaluation criteria do not allow any further differentiation for a small set of similarly qualified applications [22,26]. The applicants were informed about the possible random selection and the evaluation process thus complied with the San Francisco Declaration on Research Assessment (DORA) [27], which states that funders must be explicit about assessment criteria. There was some reservation on the random selection approach among some panel members, but acceptance grew over time. Of note, panels applied the random selection only in a few cases, in eight (6.0%) of 134 applications. In the context of the Explorer Grant scheme of the HRC-NZ, Liu et. al [28] recently reported that most applicants agreed with the use of a random selection. In this study, no negative or positive reactions to the use of random selection were received from applicants.

For peer review only - http://bmjopen.bmj.com/site/about/guidelines.xhtml

Our study has several limitations. It addressed the specific context of the SNSF Postdoc.Mobility funding scheme and results may not be generalizable to other funding instruments. The sample size was relatively small, and the study lacked statistical power, for example, to examine differences in agreement between TPM and ERB evaluation across disciplines. The two evaluation methods were not independent, since the two assessments of the panel reviewers were used for both methods. We were relying on reviewer evaluation scores which might not always perfectly reflect the quality of the proposed project, might be biased, and depend on the reviewers' previous experience with grant evaluation. However, our study design allowed us to investigate the impact of panel meetings on funding outcomes compared to an ERB approach. This study provides further insights into peer review and a modified lottery approach selection in the context of the evaluation of fellowship applications. More research on the limitations inherent in peer review and grant evaluation is urgently needed. Funders should be creative when investigating the merit of different evaluation strategies [29].

Conclusions

In conclusion, we simulated an ERB approach in the evaluation of the junior Postdoc.Mobility funding scheme at the SNSF and compared the funding outcome to the standard TPM format, which has been in use for many years. We found an overall high agreement between the two methods. Discrepancies were mainly observed in the middle group of applications that were discussed in the panel meetings. Based on the evidence that peer review has difficulties in making fine-grained differentiations between meritorious applications [20–22], we are unsure which method performs better. Our findings indicate that the ERB approach represents a viable evaluation method for the Postdoc.Mobility selection process that could save cost and time which could be invested in science and research.

For peer review only - http://bmjopen.bmj.com/site/about/guidelines.xhtml

For peer review only

Acknowledgements

We thank the Management of the SNSF Administrative Offices for approving additional resources for the conduction of this study. We also thank the SNSF Postdoc.Mobility staff of the Administrative Offices for their excellent support in implementing the additional reviewers used for the study.

Contributors

Conceived and designed the experiments: MB KR ME. Performed the experiments: MB KR. Analyzed the data: KR RH. Contributed reagents/materials/analysis tools: MB KR ME RH. Wrote the initial draft: MB. Contributed to writing: KR ME RH.

Funding

This research received no specific grant from any funding agency in the public, commercial or not-for-profit sectors.

For peer review only

For peer review only - http://bmjopen.bmj.com/site/about/guidelines.xhtml

References

1 Guthrie S, Ghiga I, Wooding S. What do we know about grant peer review in the health sciences? *F1000Res* 2018;**6**:1335. doi:10.12688/f1000research.11917.2

2 Guthrie S, Rodriguez Rincon D, McInroy G, *et al.* Measuring bias, burden and conservatism in research funding processes. *F1000Res* 2019;**8**:851. doi:10.12688/f1000research.19156.1

3 Snell RR. Menage a Quoi? Optimal Number of Peer Reviewers. *PLoS ONE* 2015;**10**:e0120838. doi:10.1371/journal.pone.0120838

4 Graves N, Barnett AG, Clarke P. Funding grant proposals for scientific research: retrospective analysis of scores by members of grant review panel. *BMJ* 2011;**343**:d4797–d4797. doi:10.1136/bmj.d4797

5 Herbert DL, Graves N, Clarke P, *et al.* Using simplified peer review processes to fund research: a prospective study. *BMJ Open* 2015;**5**:e008380. doi:10.1136/bmjopen-2015-008380

6 Bohannon J. Meeting for Peer Review at a Resort That's Virtually Free. *Science* 2011;**331**:27–27. doi:10.1126/science.331.6013.27

7 Gallo SA, Carpenter AS, Glisson SR. Teleconference versus Face-to-Face Scientific Peer Review of Grant Application: Effects on Review Outcomes. *PLoS ONE* 2013;**8**:e71693. doi:10.1371/journal.pone.0071693

8 Carpenter AS, Sullivan JH, Deshmukh A, *et al.* A retrospective analysis of the effect of discussion in teleconference and face-to-face scientific peer-review panels. *BMJ Open* 2015;**5**:e009138. doi:10.1136/bmjopen-2015-009138

9 Pier EL, Raclaw J, Nathan MJ, *et al.* Studying the Study Section: How Group Decision Making in Person and via Videoconferencing Affects the Grant Peer Review Process. 2015.https://wcer.wisc.edu/docs/working-papers/Working_Paper_No_2015_06.pdf

10 Vo NM, Quiggle GM, Wadhwani K. Comparative outcomes of face-to-face and virtual review meetings. *International Journal of Surgery Open* 2016;**4**:38–41. doi:10.1016/j.ijso.2016.07.002

11 Fogelholm M, Leppinen S, Auvinen A, *et al.* Panel discussion does not improve reliability of peer review for medical research grant proposals. *Journal of Clinical Epidemiology* 2012;**65**:47–52. doi:10.1016/j.jclinepi.2011.05.001

12 Obrecht M, Tibelius K, D'Aloisio G. Examining the value added by committee discussion in the review of applications for research awards. *res eval* 2007;**16**:70–91. doi:10.3152/095820207X223785

13 Pina DG, Hren D, Marušić A. Peer Review Evaluation Process of Marie Curie Actions under EU's Seventh Framework Programme for Research. *PLoS ONE* 2015;**10**:e0130753. doi:10.1371/journal.pone.0130753

For peer review only - http://bmjopen.bmj.com/site/about/guidelines.xhtml
For peer review only

14 Vener KJ, Feuer EJ, Gorelic L. A statistical model validating triage for the peer review process: keeping the competitive applications in the review pipeline [1]. *FASEB j* 1993;**7**:1312–9. doi:10.1096/fasebj.7.14.8224604

15 Bornmann L, Mutz R, Daniel H-D. Latent Markov modeling applied to grant peer review. *Journal of Informetrics* 2008;**2**:217–28. doi:10.1016/j.joi.2008.05.003

16 SNSF Postdoc.Mobility Regulations. http://www.snf.ch/SiteCollectionDocuments/Reglement_PM_ab2021_en.pdf

17 Cole S, Cole, Simon G. Chance and consensus in peer review. *Science* 1981;**214**:881–6. doi:10.1126/science.7302566

18 Hodgson C. How reliable is peer review? An examination of operating grant proposals simultaneously submitted to two similar peer review systems. *Journal of Clinical Epidemiology* 1997;**50**:1189–95. doi:10.1016/S0895-4356(97)00167-4

19 Clarke P, Herbert D, Graves N, *et al.* A randomized trial of fellowships for early career researchers finds a high reliability in funding decisions. *Journal of Clinical Epidemiology* 2016;**69**:147–51. doi:10.1016/j.jclinepi.2015.04.010

20 Scheiner SM, Bouchie LM. The predictive power of NSF reviewers and panels. *Frontiers in Ecology and the Environment* 2013;**11**:406–7. doi:10.1890/13.WB.017

21 Fang FC, Bowen A, Casadevall A. NIH peer review percentile scores are poorly predictive of grant productivity. *eLife* 2016;**5**:e13323. doi:10.7554/eLife.13323

22 Klaus B, del Alamo D. Talent Identification at the limits of Peer Review: an analysis of the EMBO Postdoctoral Fellowships Selection Process. Scientific Communication and Education 2018. doi:10.1101/481655

23 Health Research Council of New Zealand Explorer Grants. https://gateway.hrc.govt.nz/funding/researcher-initiated-proposals/2021-explorer-grants

24 Volkswagen Foundation Experiment! https://www.volkswagenstiftung.de/en/funding/our-funding-portfolio-at-a-glance/experiment

25 FWF 1000 Ideas Programme. https://www.fwf.ac.at/en/research-funding/fwf-programmes/1000-ideas-programme/

26 Fang FC, Casadevall A. Research Funding: the Case for a Modified Lottery. *mBio* 2016;**7**:e00422-16, /mbio/7/2/e00422-16.atom. doi:10.1128/mBio.00422-16

27 San Francisco Declaration on Research Assessment (DORA). https://sfdora.org/

28 Liu M, Choy V, Clarke P, *et al.* The acceptability of using a lottery to allocate research funding: a survey of applicants. *Res Integr Peer Rev* 2020;**5**:3. doi:10.1186/s41073-019-0089-z

For peer review only

29 Severin A, Egger M. Research on research funding: an imperative for science and society. *Br J Sports Med* 2020;:bjsports-2020-103340. doi:10.1136/bjsports-2020-103340

For peer review only

For peer review only - http://bmjopen.bmj.com/site/about/guidelines.xhtml

Figures and Tables

Figure 1. Design of the study comparing the Expert Review-Based evaluation (ERB) with the Triage-Panel Meeting (TPM) format. The ERB and the TPM were dependent in terms of the two assigned panel reviewers per application. The third reviewers were only added for the ERB, their assessments were not considered for the TPM and therefore the official funding outcome.

For peer review only

For peer review only - http://bmjopen.bmj.com/site/about/guidelines.xhtml

Table 1. Success rates by gender of applicants, by discipline and type of evaluation.

	All applicants		Women		Men	
**Discipline**	**N**	**N funded (%)**	**N**	**N funded (%)**	**N**	**N funded (%)**
**TPM**						
All disciplines	134	72 (53.7)	48	29 (60.4)	86	43 (50.0)
Humanities	16	9 (56.2)	9	4 (44.4)	7	5 (71.4)
Social Sciences	23	12 (52.2)	10	7 (70.0)	13	5 (38.5)
STEM	35	19 (54.3)	10	6 (60.0)	25	13 (52.0)
Biology	40	21 (52.5)	14	8 (57.1)	26	13 (50.0)
Medicine	20	11 (55.0)	5	4 (80.0)	15	7 (46.7)
**ERB (3 reviewers*)**						
All disciplines	134	72 (53.7)	48	27 (56.3)	86	45 (52.3)
Humanities	16	9 (56.3)	9	5 (55.6)	7	4 (57.1)
Social Sciences	23	12 (52.2)	10	6 (60.0)	13	6 (46.2)
STEM	35	19 (54.3)	10	4 (40.0)	25	15 (60.0)
Biology	40	21 (52.5)	14	8 (57.1)	26	13 (50.0)
Medicine	20	11 (55.0)	5	4 (80.0)	15	7 (46.7)
**ERB (2 reviewers[&])**						
All disciplines	134	72 (53.7)	48	25 (52.1)	86	47 (54.7)
Humanities	16	9 (56.3)	9	5 (55.6)	7	4 (57.1)
Social Sciences	23	12 (52.2)	10	6 (60.0)	13	6 (46.2)
STEM	35	19 (54.3)	10	4 (40.0)	25	15 (60.0)
Biology	40	21 (52.5)	14	7 (50.0)	26	14 (53.8)
Medicine	20	11 (55.0)	5	3 (60.0)	15	8 (53.3)

Abbreviations: N: Number of applications; STEM: Science, Technology, Engineering, Mathematics; TPM: Triage-panel meeting format; ERB: Expert review-based evaluation.

*Two of the three expert reviewers were also members of the evaluation panel.

[&]Both expert reviewers were also members of the evaluation panel.

For peer review only

For peer review only - http://bmjopen.bmj.com/site/about/guidelines.xhtml

Table 2. Agreement between the simulated expert review-based (ERB) evaluation and the triage-panel meeting (TPM) format, by discipline.

Discipline	N	Funded by TPM		Agreement (%) (95% Wald CI)
**Funded by ERB (3 reviewers*)**		Yes	No	
All disciplines	Yes	59	13	80.6
	No	13	49	(73.9-87.3)
Humanities	Yes	7	2	75.0
	No	2	5	(53.8-96.2)
Social Sciences	Yes	9	3	73.9
	No	3	8	(56.0-91.8)
STEM	Yes	15	4	77.1
	No	4	12	(63.2-91.0)
Biology	Yes	18	3	85.0
	No	3	16	(73.9-96.1)
Medicine	Yes	10	1	90.0
	No	1	8	(76.9-100)
P-value				0.58
**Funded by ERB (2 reviewers[&])**				
All disciplines	Yes	63	9	86.6
	No	9	53	(80.8-92.4)
Humanities	Yes	7	2	75.0
	No	2	5	(53.8-96.2)
Social Sciences	Yes	11	1	91.3
	No	1	10	(79.8-100)
STEM	Yes	16	3	82.9
	No	3	13	(70.4-95.4)
Biology	Yes	19	2	90.0
	No	2	17	(80.7-99.3)
Medicine	Yes	10	1	90.0
	No	1	8	(76.9-100)
P-value				0.51

Abbreviations: N: Number of applications; CI: Confidence interval; STEM: Science, Technology, Engineering, Mathematics; ERB: Expert review-based evaluation; TPM: Triage-panel meeting format.

P-values for differences in agreement across disciplines from chi-squared test.

*Two of the three expert reviewers were also members of the evaluation panel.

[&]Both expert reviewers were also members of the evaluation panel.

For peer review only - http://bmjopen.bmj.com/site/about/guidelines.xhtml
For peer review only

Table 3. Agreement between the simulated expert review-based (ERB) evaluation and the triage-panel meeting (TPM) format, by gender.

Gender		Funded by TPM		Agreement (%) (95% Wald CI)
**Funded by ERB (3 reviewers*)**		Yes	No	
Women	Yes	24	3	83.3
	No	5	16	(72.7-93.9)
Men	Yes	35	10	79.1
	No	8	33	(70.5-87.7)
P-value				0.71
**Funded by ERB (2 reviewers[&])**				
Women	Yes	24	1	87.5
	No	5	18	(78.1-96.9)
Men	Yes	39	8	86.0
	No	4	35	(78.7-93.3)
P-value				0.99

Abbreviations: N: Number of applications; CI: Confidence interval; STEM: Science, Technology, Engineering, Mathematics; ERB: Expert review-based evaluation; TPM: Triage-panel meeting format.

P-values for differences in agreement across genders from chi-squared test.

*Two of the three expert reviewers were also members of the evaluation panel.

[&]Both expert reviewers were also members of the evaluation panel.

For peer review only

For peer review only - http://bmjopen.bmj.com/site/about/guidelines.xhtml

Table 4. Agreement between the simulated expert review-based (ERB) evaluation and the triage-panel meeting (TPM) format, by triage results.

Triage result		Funded by TPM		Agreement (%) (95% Wald CI)
**Funded by ERB (3 reviewers*)**		Yes	No	
Fund (F)	Yes	36	0	97.3
	No	1	0	(92.1-100)
Discuss (D)	Yes	23	12	64.2
	No	12	20	(52.7-75.7)
Reject (R)	Yes	0	1	96.7
	No	0	29	(90.3-100)
P-value				<0.001
**Funded by ERB (2 reviewers[&])**				
Fund (F)	Yes	37	0	100
	No	0	0	
Discuss (D)	Yes	26	9	73.1
	No	9	23	(62.5-83.7)
Reject (R)	Yes	0	0	100
	No	0	30	
P-value				<0.001

Abbreviations: N: Number of applications; CI: Confidence interval; STEM: Science, Technology, Engineering, Mathematics; ERB: Expert review-based evaluation; TPM: Triage-panel meeting format.

P-values for differences in agreement across triage groups from chi-squared test.

*Two of the three expert reviewers were also members of the evaluation panel.

[&]Both expert reviewers were also members of the evaluation panel.

For peer review only

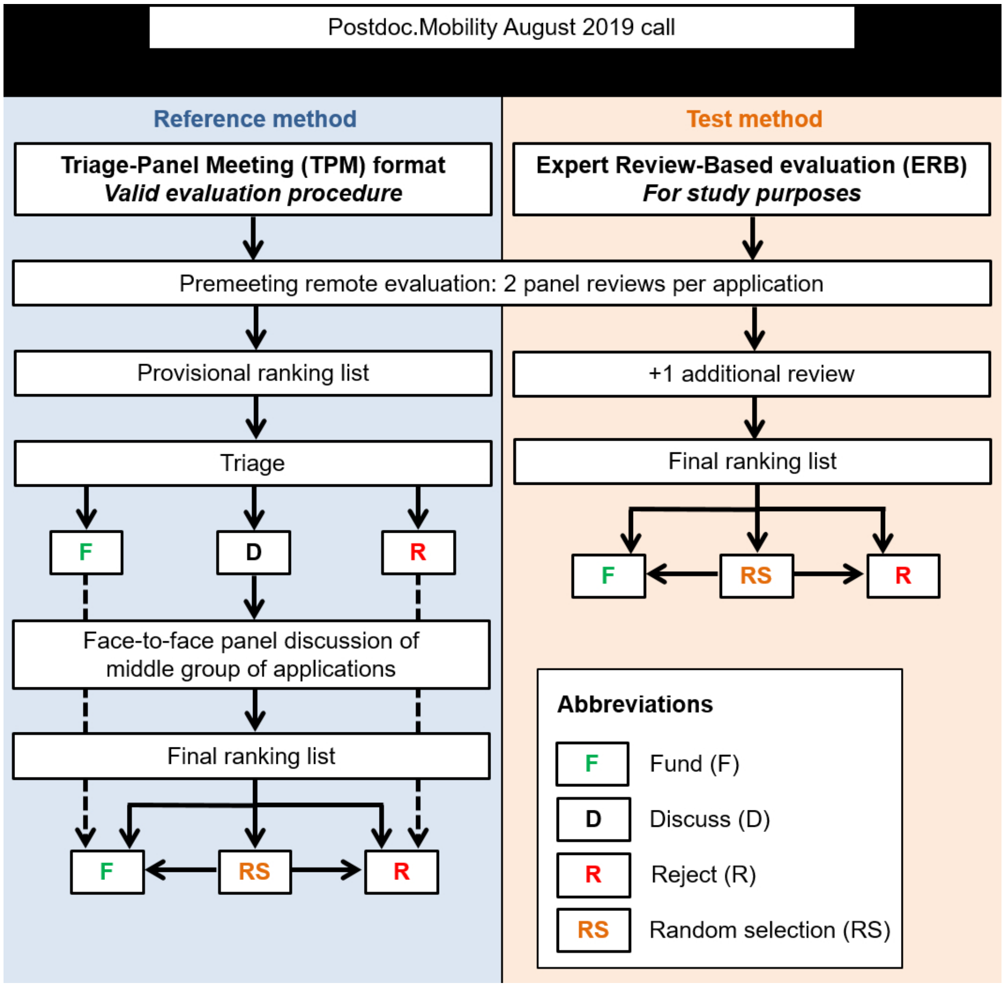

Design of the study comparing the Expert Review-Based evaluation (ERB) with the Triage-Panel Meeting (TPM) format. The ERB and the TPM were dependent in terms of the two assigned panel reviewers per application. The third reviewers were only added for the ERB, their assessments were not considered for the TPM and therefore the official funding outcome.

BMJ Open

Face-to-face panel meetings versus remote evaluation of fellowship applications: simulation study at the Swiss National Science Foundation

Journal:	*BMJ Open*
Manuscript ID	bmjopen-2020-047386.R1
Article Type:	Original research
Date Submitted by the Author:	30-Mar-2021
Complete List of Authors:	Bieri, Marco; Swiss National Science Foundation,<br>Roser, Katharina; University of Lucerne,<br>Heyard, Rachel; Swiss National Science Foundation<br>Egger, Matthias; University of Bern, Institute of Social & Preventive Medicine
**Primary Subject Heading**:	Research methods
Secondary Subject Heading:	Health policy
Keywords:	STATISTICS & RESEARCH METHODS, Health policy < HEALTH SERVICES ADMINISTRATION & MANAGEMENT, HEALTH ECONOMICS

SCHOLARONE™
Manuscripts

I, the Submitting Author has the right to grant and does grant on behalf of all authors of the Work (as defined in the below author licence), an exclusive licence and/or a non-exclusive licence for contributions from authors who are: i) UK Crown employees; ii) where BMJ has agreed a CC-BY licence shall apply, and/or iii) in accordance with the terms applicable for US Federal Government officers or employees acting as part of their official duties; on a worldwide, perpetual, irrevocable, royalty-free basis to BMJ Publishing Group Ltd ("BMJ") its licensees and where the relevant Journal is co-owned by BMJ to the co-owners of the Journal, to publish the Work in this journal and any other BMJ products and to exploit all rights, as set out in our licence.

The Submitting Author accepts and understands that any supply made under these terms is made by BMJ to the Submitting Author unless you are acting as an employee on behalf of your employer or a postgraduate student of an affiliated institution which is paying any applicable article publishing charge ("APC") for Open Access articles. Where the Submitting Author wishes to make the Work available on an Open Access basis (and intends to pay the relevant APC), the terms of reuse of such Open Access shall be governed by a Creative Commons licence – details of these licences and which Creative Commons licence will apply to this Work are set out in our licence referred to above.

Other than as permitted in any relevant BMJ Author's Self Archiving Policies, I confirm this Work has not been accepted for publication elsewhere, is not being considered for publication elsewhere and does not duplicate material already published. I confirm all authors consent to publication of this Work and authorise the granting of this licence.

For peer review only

Original Manuscript

Face-to-face panel meetings versus remote evaluation of fellowship applications: simulation study at the Swiss National Science Foundation

Marco Bieri[1¶*], Katharina Roser[1,2¶], Rachel Heyard[1], Matthias Egger[1,3,4]

[1]Swiss National Science Foundation, Berne, Switzerland

[2]Department of Health Sciences and Medicine, University of Lucerne, Lucerne, Switzerland

[3]Institute of Social and Preventive Medicine, University of Berne, Berne, Switzerland

[4]Population Health Sciences, Bristol Medical School, University of Bristol, Bristol, UK

¶These authors contributed equally to this work

[*]Corresponding author:

Marco Bieri

Swiss National Science Foundation

Wildhainweg 3

CH-3001 Berne

marco.bieri@snf.ch

Word count: 3657 words, 29 references, 4 tables, 1 figure.

For peer review only

Abstract

Objectives

To compare a cost-saving, simplified evaluation of fellowship proposals with the official evaluation by analyzing the agreement of funding decisions, and to examine the use of a lottery-based decision for proposals of similar quality.

Design

The study involved 134 junior fellowship proposals for postdoctoral research (“Postdoc.Mobility”). The official method used two panel reviewers who independently scored the application, followed by triage and discussion of selected applications in a panel. Very competitive/uncompetitive proposals were directly funded/rejected without discussion. The simplified procedure used the scores of the two panel members, with or without the score of an additional, third expert. Both methods could further use a lottery to decide on applications of similar quality close to the funding threshold. The same funding rate was applied, and the agreement between the two methods analyzed.

Setting

Swiss National Science Foundation (SNSF).

Participants

Postdoc.Mobility panel reviewers and additional expert reviewers.

Primary outcome measure

Per cent agreement between the simplified and official evaluation method with 95% confidence intervals (95% CI).

Results

The simplified procedure based on three reviews agreed in 80.6% (95% CI 73.9-87.3) with the official funding outcome. The agreement was 86.6% (95% CI 80.6-91.8) when using the two reviews of the panel members. The agreement between the two methods was lower for the group of applications discussed in the panel (64.2% and 73.1%, respectively), and higher

For peer review only

for directly funded/rejected applications (range 96.7% to 100%). The lottery was used in eight (6.0%) of 134 applications (official method), 19 (14.2%) applications (simplified, three reviewers) and 23 (17.2%) applications (simplified, two reviewers). With the simplified procedure, evaluation costs could have been halved and 31 hours of meeting time saved for the two 2019 calls.

Conclusion

Agreement between the two methods was high. The simplified procedure could represent a viable evaluation method for the Postdoc.Mobility early career instrument at the SNSF.

Strengths and limitations of this study

- The study examined the outcome between a simplified and the official evaluation procedure for junior fellowship applications for different research disciplines.
- The study discussed the agreement between the two evaluation methods in the context of the general uncertainty around peer review and estimated the costs that could have been saved with the simplified evaluation procedure.
- It is the first study to provide insight into lottery-based decisions in the context of the evaluation of junior fellowship applications.
- The study lacks statistical power because the number of applications was relatively small.
- The study addressed the specific context and evaluation of the SNSF Postdoc.Mobility funding scheme, results may thus not be generalizable to other funding programs.

For peer review only

Introduction

Peer review of grant proposals is costly and time-consuming. The burden on the scientific system is increasing, affecting funders, reviewers, and applicants [1,2]. In response, researchers have studied the review process and examined simplifications. For example, Snell [3] studied the number of reviewers and consistency of decisions and found that five evaluators represented an optimal tradeoff. Graves et al. [4] assessed the reliability of decisions made by evaluation panels of different sizes. They concluded that reliability was greatest with about ten panel members. Herbert et al. [5] compared smaller panels and shorter research proposals with the standard review procedure. The agreement was about 75% between simplified and standard procedures. As an alternative to face-to-face (FTF) panels, the use of virtual, online meetings has also been examined. Bohannon [6] reported that at the National Science Foundation (NSF) and National Institutes of Health (NIH), virtual meetings could reduce costs by one-third. Gallo et al. [7] compared teleconferencing with FTF meetings and found only few differences in the scoring of the applications. Later studies also found that virtual and FTF panels produce comparable outcomes [8–10].

With virtual formats, panel members still need to attend time-consuming meetings. Using the reviewers' written assessments without FTF or virtual panel discussions would simplify the process further. Fogelholm et al. [11] reported that results were similar when using panel consensus or the mean of reviewer scores. Obrecht et al. [12] noted that panel review changed the funding outcome of only 11% of applications. Similarly, Carpenter et al. [8] found that the impact of discussions was small, affecting the funding outcome of about 10% of applications. Pina et al. [13] studied Marie Curie Actions applications and concluded that ranking applications based on reviewer scores might work for some but not all disciplines. In the humanities, social and economic sciences, an exchange between reviewers may be particularly relevant. The triaging of applications has also been examined: after an initial screening, noncompetitive and very competitive proposals are either directly rejected or funded. Vener et al. [14] validated the triage model of the NIH and found that the likelihood of erroneously discarding a competitive proposal was very small. Bornmann et al.'s [15] findings on a multi-stage fellowship selection process also supported the use of a triage.

Mandated by the government, the Swiss National Science Foundation (SNSF) is Switzerland's foremost funding agency, supporting scientific research in all disciplines. Following changes to the career-funding portfolio, the SNSF will experience a significant increase of applications for the junior "Postdoc.Mobility" fellowship scheme, which offers

For peer review only

postdoctoral researchers a stay at a research institution abroad for up to 24 months. The scheme enables junior postdocs to deepen their scientific knowledge and increase their scientific independence during a research stay abroad. The aim of this work was to compare the evaluation of applications by expert review, triage, and discussion in an evaluation panel with expert reviews only.

Methods

Sample

We included applications submitted for the August 2019 Postdoc.Mobility fellowship call. We also included applications by Postdoc.Mobility fellows for a return grant to facilitate their return to Switzerland. Both fellowship and return grants were evaluated according to the same criteria by one of five panels: Humanities, Social Sciences, STEM (Science, Technology, Engineering and Mathematics), Biology or Medicine.

Study design

We compared funding outcomes based on the official, legally binding evaluation with a simulated, hypothetical evaluation. The official evaluation was based on the triage of applications based on expert reviews, followed by a discussion of the meritorious applications in an FTF panel: the Triage-Panel Meeting (TPM) format (Figure 1). In a first step, each proposal was independently reviewed and scored by two panel members. For the assessment, the evaluation criteria defined in the Postdoc.Mobility regulations [16] were applied. The criteria address different aspects of the applicant, the proposed research project, and the designated research location. Panel members used a 6-point scale: outstanding=6 points, excellent=5 points, very good=4 points, good=3 points, mediocre=2 points, poor=1 point. Applications were then allocated to three groups based on the ranking of the mean scores given to each proposal: Fund without further discussion (F in Figure 1), Discuss in panel meeting (D), and Reject (R). Panel members could request that applications in the F or R group are reallocated to D and discussed. In a second step, the D proposals were discussed in the FTF panel meeting, ranked and funded or rejected. Random Selection (RS in Figure 1) could be used to fund or reject proposals of similar quality close to the funding threshold if the panel could not reach a decision. Funding decisions were based on the standard two-stage method, which included FTF panel meetings (TPM).

For peer review only - http://bmjopen.bmj.com/site/about/guidelines.xhtml

The simulated alternative procedure consisted only of the first step, i.e., was entirely based on the ranking of proposals based on the expert reviews: the Expert Review-Based (ERB) evaluation. In addition to the two panel members, a third expert reviewer who was not a member of the panel assessed the proposal. The same 6-point scale was used. The proposals were then allocated to one of three groups based on the mean scores (F, RS, and R in Figure 1). Random selection was used whenever the funding line went through a group of two or more applications with identical scores. The funding rate of the TPM was applied to the simulated ERB method.

Data analysis

To determine the agreement between the two evaluation methods, we used 2x2 contingency tables. We calculated the simple agreement with 95% confidence intervals (CI), which were generated using a bootstrap algorithm [5]. We also examined the agreement between the TPM and the ERB approach using only the assessments from the two panel members, thus excluding the assessment from the third reviewer. We calculated discipline- and gender-specific levels of agreement. We used chi-squared tests for categorical data to test whether the agreement differed between these mutually exclusive groups.

Costs

We determined the costs related to the evaluation. The costs comprised expenses related to the scientific assessment of the individual applications and the panel meetings. The SNSF compensates panel reviewers with USD 275 per scientific assessment. Panel reviewers further receive a meeting allowance of up to USD 550 depending on the duration of the meeting. Further, the SNSF reimburses travel expenses and accommodation costs. The five panels included 96 members and met twice in 2019.

Ethics approval

The Ethics Committee of the Canton of Berne confirmed that the study does not fall under the Federal Act on Research involving Human Beings. No reviewer, applicant or application can be identified from this study.

Patient and Public Involvement

For peer review only - http://bmjopen.bmj.com/site/about/guidelines.xhtml
For peer review only

This analysis was based on expert review reports submitted to the Swiss National Science Foundation. No patients were involved in developing the research question, outcome measures and overall design of the study.

For peer review only

For peer review only - http://bmjopen.bmj.com/site/about/guidelines.xhtml

Results

Study sample and success rates

The sample consisted of 134 applications, including 124 fellowship applications and ten requests for a return grant. The mean age of applicants was 32.7 years (SD 3.2 years) in men and 33.5 years (SD 2.8 years) in women. Each reviewer received a mean of 2.5 (SD 1.4) applications to evaluate.

Table 1 shows the summary statistics of applications and success rates across disciplines, genders and the three evaluation methods: the legally binding TPM format and the simulated ERB evaluations with three or two reviewers. Most applications came from biology, followed by the STEM disciplines and the social sciences. Almost two-thirds of applications came from men. With TPM, success rates were slightly higher in women (60.4%) than in men (50.0%). This was driven by the middle group of applications that were discussed in the panels, where the success rates of women overall was 66.7% (24 of 67 applicants were women in this group). Success rates were similar across disciplines, ranging from 56.2% in the humanities to 52.2% in the social sciences. By design, overall success rates were the same with the ERB evaluations; however, the difference between genders was smaller with ERB than with TPM (Table 1).

Agreement between evaluation by ERB or TPM

Comparing the ERB evaluation based on three reviewers with the standard TPM format, the agreement overall was 80.6% (95% CI 73.9-87.3). The agreement was highest in the Medicine panel (90.0%; CI 75-100), and lowest in the Social Sciences panel (73.9%; CI 56.5-91.3). However, the statistical evidence for differences in agreement between panels was weak (*P*=0.58, Table 2). As expected, the agreement was higher when comparing the ERB evaluation based on the two panel members with TPM. Overall, for two reviews, the agreement was 86.6% (95% CI 80.6-91.8). It ranged from 75.0% (CI 50.0-93.8) in the Humanities panel to 91.3% (CI 78.3-100) in the Social Sciences panel. Again, there was no evidence for differences in agreement between panels (*P*=0.51). Both for ERB evaluation with three and two reviewers, the agreement was slightly higher for women than for men (*P*>0.70, Table 3).

In Table 4, we calculated agreement separately for the triage categories: Fund (F), Discuss (D), Reject (R). With the ERB evaluation based on three reviewers, agreements for F

For peer review only - http://bmjopen.bmj.com/site/about/guidelines.xhtml

and R were close to 100% (97.3% and 96.7%, respectively) but considerably lower for D: 64.2% (95% CI 52.2-76.1), with $P<0.001$ for differences in agreement across categories. For ERB evaluation with two reviewers (the two panel members), the agreement was 100% for F and R, but 73.1% (95% CI 62.7- 83.6) for D, with $P<0.001$ for differences in agreement.

Random selection in TPM and ERB evaluation

With the standard TPM evaluation, only eight (11.9%) of the 67 applicants in the D group, or eight (6.0%) of 134 applicants were entered into a lottery of whom four were funded. With the simulated ERB evaluation based on three reviewers, 19 (14.2%) of the 134 applicants would have entered the lottery, and with the ERB with two reviewers 23 (17.2%) applications would have been subjected to random selection.

Cost and time savings

We determined the resources that could be saved with the use of an ERB evaluation compared to the TPM. By comparison with the current valid TPM evaluation procedure for the Postdoc.Mobility, we calculated that about USD 91,000 related to the holding of meetings could have been saved if an ERB evaluation had been used for the two Postdoc.Mobility calls in 2019. This saving corresponds to 55% of total costs. Moreover, the holding of all panel sessions in 2019 amounted to 31 meeting hours, a significant workload that could have been avoided using the ERB approach. Lastly, the funding decisions could have been communicated at least one month earlier with ERB, reducing the time to notification by about 20% compared to TPM.

For peer review only - http://bmjopen.bmj.com/site/about/guidelines.xhtml

Discussion

In this comparative study of the evaluation of early-career funding applications, we found that the simulated funding outcomes of a simplified, expert review-based (ERB) approach agreed well with the official funding outcomes based on the standard, time-tested triage and panel meeting (TPM) format. Applications for fellowships covered a wide range of disciplines, from the humanities and social sciences to STEM, biology and medicine. The agreement was very high for proposals which, in the TPM evaluation, were either allocated to the Fund or Reject categories, but lower in the middle category of proposals that were discussed by the panels. More applicants entered the lottery with the simplified ERB approach than with TPM evaluation. Finally, the simplified ERB evaluation approach was associated with a substantial reduction in costs. Overall, our results support the notion that a sound evaluation of early-career funding applications is possible with an ERB approach.

Although panel review is considered as a "de facto" standard, the consistency of decisions from panels has been shown to be limited. For example, previous work by Cole [17], Hodgson [18], Fogelholm [11] and Clarke [19] found an agreement of 65% to 83% between two independent panels evaluating the same set of applications. Thus, in these studies, the funding outcome also depended on the panel that evaluated the application, and not only on the scientific content. Against this background, the agreement of over 80% between ERB and TPM in this study is remarkable. Among the different discipline-specific review panels, our results showed a slightly lower agreement in the humanities and social sciences compared to life sciences and medicine. These differences did not reach conventional levels of statistical significance but were in line with previous findings reported by Pina et al. [13].

In the middle group of applications based on the triage step of TPM, the agreement was lower; 64% with three reviewers and 73% with the two reviewers. This is not surprising considering the results from previous studies that suggest that peer review has difficulties in discriminating between applications that are neither clearly excellent nor clearly noncompetitive [20–22]. Agreement between ERB and TPM was also generally lower with ERB using three reviewers than with ERB with two reviewers. An additional reviewer may introduce a different viewpoint. Also, the third reviewer was not a member of the corresponding panel, and not involved in previous panel discussions, which have led to some degree of calibration between assessments of panel members. Such calibration is more difficult to achieve with a remote, ERB approach. However, information and briefing sessions

For peer review only - http://bmjopen.bmj.com/site/about/guidelines.xhtml

could be held to compensate for the lack of FTF panel meetings. Of note, previous studies reported that reviewers appreciated the social aspects and the camaraderie in FTF settings and that physical meetings are important for building trust among the evaluators [8,9].

We found that the panel discussions in the TPM format resulted in higher success rates for women compared to the ERB format. Gender equality is a key concern at the SNSF, which is committed to promoting women in research. The panels will have been aware of the under-representation of female researchers in certain areas, for example, the STEM disciplines, and the SNSF's agenda to promote women. It is, therefore, possible that during the panel deliberations and for funding decisions, the gender of applicants was taken into account in addition to the quality of the proposal.

We estimated that about USD 91,000 could have been saved for the two Postdoc.Mobility calls in 2019 if they had been evaluated by ERB rather than by TPM. The meeting costs represented about 55% of the total evaluation costs. In other words, the ERB evaluation based on the two panel reviewers would have cut the expenses by more than half. The experience described here with the junior Postdoc.Mobility fellowship scheme indicates that substantial cost savings could also result from simplifications in the evaluation of other funding instruments at the SNSF. Also, ERB would have reduced the time to communication of funding decisions, and unsuccessful applicants could thus have planned their next steps earlier. However, any such changes need to be considered carefully. The quality of the evaluation should not be allowed to be compromised because costs may be reduced.

To the best of our knowledge, the Health Research Council of New Zealand (HRC-NZ) [23], the Volkswagen Foundation [24], and recently the Austrian Research Fund FWF [25] are the only funders that have used or examined the use of a random selection element in the evaluation process of funding instruments, with a focus on transformative research or unconventional research ideas. The random selection for decisions on applications close to the funding threshold could avoid bias if evaluation criteria do not allow any further differentiation for a small set of similarly qualified applications [22,26]. The applicants were informed about the possible random selection and the evaluation process thus complied with the San Francisco Declaration on Research Assessment (DORA) [27], which states that funders must be explicit about assessment criteria. In this context, evaluation criteria could be weighted, or additional strategic criteria be used in the selection process if defined a priori and communicated to applicants. However, weighting or additional criteria could also lead to tied applications and thus require a lottery decision. There was some reservation on the random selection approach among some panel members, but acceptance grew over time. Of note,

For peer review only - http://bmjopen.bmj.com/site/about/guidelines.xhtml
For peer review only

panels applied the random selection only in a few cases, in eight (6.0%) of 134 applications. In the context of the Explorer Grant scheme of the HRC-NZ, Liu et. al [28] recently reported that most applicants agreed with the use of a random selection. In this study, the SNSF received a few unsolicited questions about the procedure but otherwise no negative or positive reactions to the use of random selection were received from applicants.

Our study has several limitations. It addressed the specific context of the SNSF Postdoc.Mobility funding scheme and results may not be generalizable to other funding instruments. The sample size was relatively small, and the study lacked statistical power, for example, to examine differences in agreement between TPM and ERB evaluation across disciplines. The two evaluation methods were not independent, since the two assessments of the panel reviewers were used for both methods. We were relying on reviewer evaluation scores which might not always perfectly reflect the quality of the proposed project, might be biased, and depend on the reviewers' previous experience with grant evaluation. However, our study design allowed us to investigate the impact of panel meetings on funding outcomes compared to an ERB approach. This study provides further insights into peer review and a modified lottery approach selection in the context of the evaluation of fellowship applications. More research on the limitations inherent in peer review and grant evaluation is urgently needed. Funders should be creative when investigating the merit of different evaluation strategies [29].

Conclusions

In conclusion, we simulated an ERB approach in the evaluation of the junior Postdoc.Mobility funding scheme at the SNSF and compared the funding outcome to the standard TPM format, which has been in use for many years. We found an overall high agreement between the two methods. Discrepancies were mainly observed in the middle group of applications that were discussed in the panel meetings. Based on the evidence that peer review has difficulties in making fine-grained differentiations between meritorious applications [20–22], we are unsure which method performs better. Our findings indicate that the ERB approach represents a viable evaluation method for the Postdoc.Mobility selection process that could save cost and time which could be invested in science and research.

For peer review only - http://bmjopen.bmj.com/site/about/guidelines.xhtml

Acknowledgements

We thank the Management of the SNSF Administrative Offices for helpful comments on the design of this study. We also thank the SNSF Postdoc.Mobility staff of the Administrative Offices for their excellent support in implementing the additional reviewers used for the study.

Contributors

Conceived and designed the experiments: MB KR ME. Performed the experiments: MB KR. Analyzed the data: KR RH. Contributed reagents/materials/analysis tools: MB KR ME RH. Wrote the initial draft: MB. Contributed to writing: KR ME RH.

Funding

This research was supported by institutional resources of the SNSF. ME was supported by a special project grant (grant No. 189498).

Competing interests

None declared.

For peer review only

References

1 Guthrie S, Ghiga I, Wooding S. What do we know about grant peer review in the health sciences? *F1000Res* 2018;**6**:1335. doi:10.12688/f1000research.11917.2

2 Guthrie S, Rodriguez Rincon D, McInroy G, *et al.* Measuring bias, burden and conservatism in research funding processes. *F1000Res* 2019;**8**:851. doi:10.12688/f1000research.19156.1

3 Snell RR. Menage a Quoi? Optimal Number of Peer Reviewers. *PLoS ONE* 2015;**10**:e0120838. doi:10.1371/journal.pone.0120838

4 Graves N, Barnett AG, Clarke P. Funding grant proposals for scientific research: retrospective analysis of scores by members of grant review panel. *BMJ* 2011;**343**:d4797–d4797. doi:10.1136/bmj.d4797

5 Herbert DL, Graves N, Clarke P, *et al.* Using simplified peer review processes to fund research: a prospective study. *BMJ Open* 2015;**5**:e008380. doi:10.1136/bmjopen-2015-008380

6 Bohannon J. Meeting for Peer Review at a Resort That's Virtually Free. *Science* 2011;**331**:27–27. doi:10.1126/science.331.6013.27

7 Gallo SA, Carpenter AS, Glisson SR. Teleconference versus Face-to-Face Scientific Peer Review of Grant Application: Effects on Review Outcomes. *PLoS ONE* 2013;**8**:e71693. doi:10.1371/journal.pone.0071693

8 Carpenter AS, Sullivan JH, Deshmukh A, *et al.* A retrospective analysis of the effect of discussion in teleconference and face-to-face scientific peer-review panels. *BMJ Open* 2015;**5**:e009138. doi:10.1136/bmjopen-2015-009138

9 Pier EL, Raclaw J, Nathan MJ, *et al.* Studying the Study Section: How Group Decision Making in Person and via Videoconferencing Affects the Grant Peer Review Process. 2015.https://wcer.wisc.edu/docs/working-papers/Working_Paper_No_2015_06.pdf

10 Vo NM, Quiggle GM, Wadhwani K. Comparative outcomes of face-to-face and virtual review meetings. *International Journal of Surgery Open* 2016;**4**:38–41. doi:10.1016/j.ijso.2016.07.002

11 Fogelholm M, Leppinen S, Auvinen A, *et al.* Panel discussion does not improve reliability of peer review for medical research grant proposals. *Journal of Clinical Epidemiology* 2012;**65**:47–52. doi:10.1016/j.jclinepi.2011.05.001

12 Obrecht M, Tibelius K, D'Aloisio G. Examining the value added by committee discussion in the review of applications for research awards. *res eval* 2007;**16**:70–91. doi:10.3152/095820207X223785

13 Pina DG, Hren D, Marušić A. Peer Review Evaluation Process of Marie Curie Actions under EU's Seventh Framework Programme for Research. *PLoS ONE* 2015;**10**:e0130753. doi:10.1371/journal.pone.0130753

For peer review only - http://bmjopen.bmj.com/site/about/guidelines.xhtml

14 Vener KJ, Feuer EJ, Gorelic L. A statistical model validating triage for the peer review process: keeping the competitive applications in the review pipeline [1]. *FASEB j* 1993;**7**:1312–9. doi:10.1096/fasebj.7.14.8224604

15 Bornmann L, Mutz R, Daniel H-D. Latent Markov modeling applied to grant peer review. *Journal of Informetrics* 2008;**2**:217–28. doi:10.1016/j.joi.2008.05.003

16 SNSF Postdoc.Mobility Regulations. http://www.snf.ch/SiteCollectionDocuments/Reglement_PM_ab2021_en.pdf

17 Cole S, Cole, Simon G. Chance and consensus in peer review. *Science* 1981;**214**:881–6. doi:10.1126/science.7302566

18 Hodgson C. How reliable is peer review? An examination of operating grant proposals simultaneously submitted to two similar peer review systems. *Journal of Clinical Epidemiology* 1997;**50**:1189–95. doi:10.1016/S0895-4356(97)00167-4

19 Clarke P, Herbert D, Graves N, *et al.* A randomized trial of fellowships for early career researchers finds a high reliability in funding decisions. *Journal of Clinical Epidemiology* 2016;**69**:147–51. doi:10.1016/j.jclinepi.2015.04.010

20 Scheiner SM, Bouchie LM. The predictive power of NSF reviewers and panels. *Frontiers in Ecology and the Environment* 2013;**11**:406–7. doi:10.1890/13.WB.017

21 Fang FC, Bowen A, Casadevall A. NIH peer review percentile scores are poorly predictive of grant productivity. *eLife* 2016;**5**:e13323. doi:10.7554/eLife.13323

22 Klaus B, del Alamo D. Talent Identification at the limits of Peer Review: an analysis of the EMBO Postdoctoral Fellowships Selection Process. Scientific Communication and Education 2018. doi:10.1101/481655

23 Health Research Council of New Zealand Explorer Grants. https://gateway.hrc.govt.nz/funding/researcher-initiated-proposals/2021-explorer-grants

24 Volkswagen Foundation Experiment! https://www.volkswagenstiftung.de/en/funding/our-funding-portfolio-at-a-glance/experiment

25 FWF 1000 Ideas Programme. https://www.fwf.ac.at/en/research-funding/fwf-programmes/1000-ideas-programme/

26 Fang FC, Casadevall A. Research Funding: the Case for a Modified Lottery. *mBio* 2016;**7**:e00422-16, /mbio/7/2/e00422-16.atom. doi:10.1128/mBio.00422-16

27 San Francisco Declaration on Research Assessment (DORA). https://sfdora.org/

28 Liu M, Choy V, Clarke P, *et al.* The acceptability of using a lottery to allocate research funding: a survey of applicants. *Res Integr Peer Rev* 2020;**5**:3. doi:10.1186/s41073-019-0089-z

For peer review only

29 Severin A, Egger M. Research on research funding: an imperative for science and society. *Br J Sports Med* 2020;:bjsports-2020-103340. doi:10.1136/bjsports-2020-103340

For peer review only

Figures and Tables

Figure 1. Design of the study comparing the Expert Review-Based evaluation (ERB) with the Triage-Panel Meeting (TPM) format. The ERB and the TPM were dependent in terms of the two assigned panel reviewers per application. The third reviewers were only added for the ERB, their assessments were not considered for the TPM and therefore the official funding outcome.

For peer review only

For peer review only - http://bmjopen.bmj.com/site/about/guidelines.xhtml

Table 1. Success rates by gender of applicants, by discipline and type of evaluation.

	All applicants		Women		Men	
**Discipline**	**N**	**N funded (%)**	**N**	**N funded (%)**	**N**	**N funded (%)**
**TPM**						
All disciplines	134	72 (53.7)	48	29 (60.4)	86	43 (50.0)
Humanities	16	9 (56.2)	9	4 (44.4)	7	5 (71.4)
Social Sciences	23	12 (52.2)	10	7 (70.0)	13	5 (38.5)
STEM	35	19 (54.3)	10	6 (60.0)	25	13 (52.0)
Biology	40	21 (52.5)	14	8 (57.1)	26	13 (50.0)
Medicine	20	11 (55.0)	5	4 (80.0)	15	7 (46.7)
**ERB (3 reviewers*)**						
All disciplines	134	72 (53.7)	48	27 (56.3)	86	45 (52.3)
Humanities	16	9 (56.3)	9	5 (55.6)	7	4 (57.1)
Social Sciences	23	12 (52.2)	10	6 (60.0)	13	6 (46.2)
STEM	35	19 (54.3)	10	4 (40.0)	25	15 (60.0)
Biology	40	21 (52.5)	14	8 (57.1)	26	13 (50.0)
Medicine	20	11 (55.0)	5	4 (80.0)	15	7 (46.7)
**ERB (2 reviewers[&])**						
All disciplines	134	72 (53.7)	48	25 (52.1)	86	47 (54.7)
Humanities	16	9 (56.3)	9	5 (55.6)	7	4 (57.1)
Social Sciences	23	12 (52.2)	10	6 (60.0)	13	6 (46.2)
STEM	35	19 (54.3)	10	4 (40.0)	25	15 (60.0)
Biology	40	21 (52.5)	14	7 (50.0)	26	14 (53.8)
Medicine	20	11 (55.0)	5	3 (60.0)	15	8 (53.3)

Abbreviations: N: Number of applications; STEM: Science, Technology, Engineering, Mathematics; TPM: Triage-panel meeting format; ERB: Expert review-based evaluation.

*Two of the three expert reviewers were also members of the evaluation panel.

[&]Both expert reviewers were also members of the evaluation panel.

For peer review only

Table 2. Agreement between the simulated expert review-based (ERB) evaluation and the triage-panel meeting (TPM) format, by discipline.

Discipline	N	Funded by TPM		Agreement (%) (95% CI)
**Funded by ERB (3 reviewers*)**		Yes	No	
All disciplines	Yes	59	13	80.6
	No	13	49	(73.9-87.3)
Humanities	Yes	7	2	75.0
	No	2	5	(50.0-93.8)
Social Sciences	Yes	9	3	73.9
	No	3	8	(56.5-91.3)
STEM	Yes	15	4	77.1
	No	4	12	(62.9-91.4)
Biology	Yes	18	3	85.0
	No	3	16	(72.5-95)
Medicine	Yes	10	1	90.0
	No	1	8	(75-100)
P-value				0.58
**Funded by ERB (2 reviewers&)**				
All disciplines	Yes	63	9	86.6
	No	9	53	(80.6-91.8)
Humanities	Yes	7	2	75.0
	No	2	5	(50.0-93.8)
Social Sciences	Yes	11	1	91.3
	No	1	10	(78.3-100)
STEM	Yes	16	3	82.9
	No	3	13	(68.6-94.3)
Biology	Yes	19	2	90.0
	No	2	17	(80-97.5)
Medicine	Yes	10	1	90.0
	No	1	8	(75-100)
P-value				0.51

Abbreviations: N: Number of applications; CI: Confidence interval; STEM: Science, Technology, Engineering, Mathematics; ERB: Expert review-based evaluation; TPM: Triage-panel meeting format.

P-values for differences in agreement across disciplines from chi-squared test.

*Two of the three expert reviewers were also members of the evaluation panel.

&Both expert reviewers were also members of the evaluation panel.

For peer review only - http://bmjopen.bmj.com/site/about/guidelines.xhtml

Table 3. Agreement between the simulated expert review-based (ERB) evaluation and the triage-panel meeting (TPM) format, by gender.

Gender		Funded by TPM		Agreement (%) (95% CI)
**Funded by ERB (3 reviewers*)**		Yes	No	
Women	Yes	24	3	83.3
	No	5	16	(72.9-93.8)
Men	Yes	35	10	79.1
	No	8	33	(69.8-87.2)
P-value				0.71
**Funded by ERB (2 reviewers&)**				
Women	Yes	24	1	87.5
	No	5	18	(77.1-95.8)
Men	Yes	39	8	86.0
	No	4	35	(77.9-93.0)
P-value				0.99

Abbreviations: N: Number of applications; CI: Confidence interval; STEM: Science, Technology, Engineering, Mathematics; ERB: Expert review-based evaluation; TPM: Triage-panel meeting format.

P-values for differences in agreement across genders from chi-squared test.

*Two of the three expert reviewers were also members of the evaluation panel.

[&]Both expert reviewers were also members of the evaluation panel.

For peer review only

Table 4. Agreement between the simulated expert review-based (ERB) evaluation and the triage-panel meeting (TPM) format, by triage results.

Triage result		Funded by TPM		Agreement (%) (95% CI)
**Funded by ERB (3 reviewers*)**		Yes	No	
Fund (F)	Yes	36	0	97.3
	No	1	0	(91.9-100)
Discuss (D)	Yes	23	12	64.2
	No	12	20	(52.2-76.1)
Reject (R)	Yes	0	1	96.7
	No	0	29	(90.0-100)
P-value				<0.001
**Funded by ERB (2 reviewers[&])**				
Fund (F)	Yes	37	0	100
	No	0	0	
Discuss (D)	Yes	26	9	73.1
	No	9	23	(62.7-83.6)
Reject (R)	Yes	0	0	100
	No	0	30	
P-value				<0.001

Abbreviations: N: Number of applications; CI: Confidence interval; STEM: Science, Technology, Engineering, Mathematics; ERB: Expert review-based evaluation; TPM: Triage-panel meeting format.

P-values for differences in agreement across triage groups from chi-squared test.

*Two of the three expert reviewers were also members of the evaluation panel.

[&]Both expert reviewers were also members of the evaluation panel.

For peer review only

For peer review only - http://bmjopen.bmj.com/site/about/guidelines.xhtml

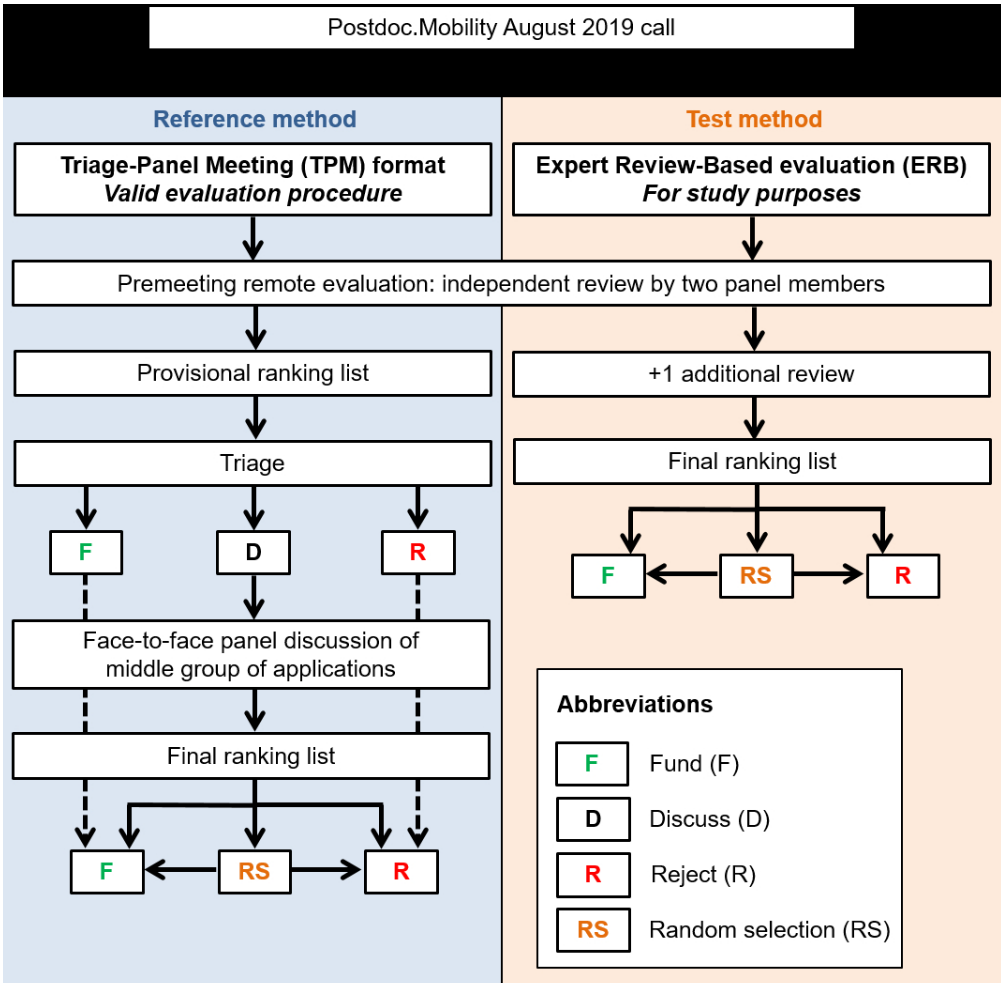

Figure 1. Design of the study comparing the Expert Review-Based evaluation (ERB) with the Triage-Panel Meeting (TPM) format. The ERB and the TPM were dependent in terms of the two assigned panel reviewers per application. The third reviewers were only added for the ERB, their assessments were not considered for the TPM and therefore the official funding outcome.

BMJ Open

Face-to-face panel meetings versus remote evaluation of fellowship applications: simulation study at the Swiss National Science Foundation

Journal:	*BMJ Open*
Manuscript ID	bmjopen-2020-047386.R2
Article Type:	Original research
Date Submitted by the Author:	08-Apr-2021
Complete List of Authors:	Bieri, Marco; Swiss National Science Foundation,<br>Roser, Katharina; University of Lucerne,<br>Heyard, Rachel; Swiss National Science Foundation<br>Egger, Matthias; University of Bern, Institute of Social & Preventive Medicine
**Primary Subject Heading**:	Research methods
Secondary Subject Heading:	Health policy
Keywords:	STATISTICS & RESEARCH METHODS, Health policy < HEALTH SERVICES ADMINISTRATION & MANAGEMENT, HEALTH ECONOMICS

SCHOLARONE™
Manuscripts

I, the Submitting Author has the right to grant and does grant on behalf of all authors of the Work (as defined in the below author licence), an exclusive licence and/or a non-exclusive licence for contributions from authors who are: i) UK Crown employees; ii) where BMJ has agreed a CC-BY licence shall apply, and/or iii) in accordance with the terms applicable for US Federal Government officers or employees acting as part of their official duties; on a worldwide, perpetual, irrevocable, royalty-free basis to BMJ Publishing Group Ltd ("BMJ") its licensees and where the relevant Journal is co-owned by BMJ to the co-owners of the Journal, to publish the Work in this journal and any other BMJ products and to exploit all rights, as set out in our licence.

The Submitting Author accepts and understands that any supply made under these terms is made by BMJ to the Submitting Author unless you are acting as an employee on behalf of your employer or a postgraduate student of an affiliated institution which is paying any applicable article publishing charge ("APC") for Open Access articles. Where the Submitting Author wishes to make the Work available on an Open Access basis (and intends to pay the relevant APC), the terms of reuse of such Open Access shall be governed by a Creative Commons licence – details of these licences and which Creative Commons licence will apply to this Work are set out in our licence referred to above.

Other than as permitted in any relevant BMJ Author's Self Archiving Policies, I confirm this Work has not been accepted for publication elsewhere, is not being considered for publication elsewhere and does not duplicate material already published. I confirm all authors consent to publication of this Work and authorise the granting of this licence.

For peer review only

Original Manuscript

Face-to-face panel meetings versus remote evaluation of fellowship applications: simulation study at the Swiss National Science Foundation

Marco Bieri[1¶*], Katharina Roser[1,2¶], Rachel Heyard[1], Matthias Egger[1,3,4]

[1]Swiss National Science Foundation, Berne, Switzerland

[2]Department of Health Sciences and Medicine, University of Lucerne, Lucerne, Switzerland

[3]Institute of Social and Preventive Medicine, University of Berne, Berne, Switzerland

[4]Population Health Sciences, Bristol Medical School, University of Bristol, Bristol, UK

¶These authors contributed equally to this work

*Corresponding author:

Marco Bieri

Swiss National Science Foundation

Wildhainweg 3

CH-3001 Berne

marco.bieri@snf.ch

Word count: 3683 words, 29 references, 4 tables, 1 figure.

For peer review only - http://bmjopen.bmj.com/site/about/guidelines.xhtml

Abstract

Objectives

To trial a simplified, time and cost-saving method for remote evaluation of fellowship applications and compare this with existing panel review processes by analyzing concordance between funding decisions, and the use of a lottery-based decision method for proposals of similar quality.

Design

The study involved 134 junior fellowship proposals for postdoctoral research ("Postdoc.Mobility"). The official method used two panel reviewers who independently scored the application, followed by triage and discussion of selected applications in a panel. Very competitive/uncompetitive proposals were directly funded/rejected without discussion. The simplified procedure used the scores of the two panel members, with or without the score of an additional, third expert. Both methods could further use a lottery to decide on applications of similar quality close to the funding threshold. The same funding rate was applied, and the agreement between the two methods analyzed.

Setting

Swiss National Science Foundation (SNSF).

Participants

Postdoc.Mobility panel reviewers and additional expert reviewers.

Primary outcome measure

Per cent agreement between the simplified and official evaluation method with 95% confidence intervals (95% CI).

Results

The simplified procedure based on three reviews agreed in 80.6% (95% CI 73.9-87.3) with the official funding outcome. The agreement was 86.6% (95% CI 80.6-91.8) when using the

For peer review only - http://bmjopen.bmj.com/site/about/guidelines.xhtml

two reviews of the panel members. The agreement between the two methods was lower for the group of applications discussed in the panel (64.2% and 73.1%, respectively), and higher for directly funded/rejected applications (range 96.7% to 100%). The lottery was used in eight (6.0%) of 134 applications (official method), 19 (14.2%) applications (simplified, three reviewers) and 23 (17.2%) applications (simplified, two reviewers). With the simplified procedure, evaluation costs could have been halved and 31 hours of meeting time saved for the two 2019 calls.

Conclusion

Agreement between the two methods was high. The simplified procedure could represent a viable evaluation method for the Postdoc.Mobility early career instrument at the SNSF.

For peer review only

Strengths and limitations of this study

- The study examined the outcome between a simplified and the official evaluation procedure for junior fellowship applications for different research disciplines.
- The study discussed the agreement between the two evaluation methods in the context of the general uncertainty around peer review and estimated the costs that could have been saved with the simplified evaluation procedure.
- It is the first study to provide insight into lottery-based decisions in the context of the evaluation of junior fellowship applications.
- The study lacks statistical power because the number of applications was relatively small.
- The study addressed the specific context and evaluation of the SNSF Postdoc.Mobility funding scheme, results may thus not be generalizable to other funding programs.

For peer review only - http://bmjopen.bmj.com/site/about/guidelines.xhtml

Introduction

Peer review of grant proposals is costly and time-consuming. The burden on the scientific system is increasing, affecting funders, reviewers, and applicants [1,2]. In response, researchers have studied the review process and examined simplifications. For example, Snell [3] studied the number of reviewers and consistency of decisions and found that five evaluators represented an optimal tradeoff. Graves et al. [4] assessed the reliability of decisions made by evaluation panels of different sizes. They concluded that reliability was greatest with about ten panel members. Herbert et al. [5] compared smaller panels and shorter research proposals with the standard review procedure. The agreement was about 75% between simplified and standard procedures. As an alternative to face-to-face (FTF) panels, the use of virtual, online meetings has also been examined. Bohannon [6] reported that at the National Science Foundation (NSF) and National Institutes of Health (NIH), virtual meetings could reduce costs by one-third. Gallo et al. [7] compared teleconferencing with FTF meetings and found only few differences in the scoring of the applications. Later studies also found that virtual and FTF panels produce comparable outcomes [8–10].

With virtual formats, panel members still need to attend time-consuming meetings. Using the reviewers' written assessments without FTF or virtual panel discussions would simplify the process further. Fogelholm et al. [11] reported that results were similar when using panel consensus or the mean of reviewer scores. Obrecht et al. [12] noted that panel review changed the funding outcome of only 11% of applications. Similarly, Carpenter et al. [8] found that the impact of discussions was small, affecting the funding outcome of about 10% of applications. Pina et al. [13] studied Marie Curie Actions applications and concluded that ranking applications based on reviewer scores might work for some but not all disciplines. In the humanities, social and economic sciences, an exchange between reviewers may be particularly relevant. The triaging of applications has also been examined: after an initial screening, noncompetitive and very competitive proposals are either directly rejected or funded. Vener et al. [14] validated the triage model of the NIH and found that the likelihood of erroneously discarding a competitive proposal was very small. Bornmann et al.'s [15] findings on a multi-stage fellowship selection process also supported the use of a triage.

Mandated by the government, the Swiss National Science Foundation (SNSF) is Switzerland's foremost funding agency, supporting scientific research in all disciplines. Following changes to the career-funding portfolio, the SNSF will experience a significant increase of applications for the junior "Postdoc.Mobility" fellowship scheme, which offers

For peer review only - http://bmjopen.bmj.com/site/about/guidelines.xhtml

postdoctoral researchers a stay at a research institution abroad for up to 24 months. The scheme enables junior postdocs to deepen their scientific knowledge and increase their scientific independence during a research stay abroad. The aim of this work was to compare the evaluation of applications by expert review, triage, and discussion in an evaluation panel with expert reviews only.

Methods

Sample

We included applications submitted for the August 2019 Postdoc.Mobility fellowship call. We also included applications by Postdoc.Mobility fellows for a return grant to facilitate their return to Switzerland. Both fellowship and return grants were evaluated according to the same criteria by one of five panels: Humanities, Social Sciences, STEM (Science, Technology, Engineering and Mathematics), Biology or Medicine.

Study design

We compared funding outcomes based on the official, legally binding evaluation with a simulated, hypothetical evaluation. The official evaluation was based on the triage of applications based on expert reviews, followed by a discussion of the meritorious applications in an FTF panel: the Triage-Panel Meeting (TPM) format (Figure 1). In a first step, each proposal was independently reviewed and scored by two panel members. For the assessment, the evaluation criteria defined in the Postdoc.Mobility regulations [16] were applied. The criteria address different aspects of the applicant, the proposed research project, and the designated research location. Panel members used a 6-point scale: outstanding=6 points, excellent=5 points, very good=4 points, good=3 points, mediocre=2 points, poor=1 point. Applications were then allocated to three groups based on the ranking of the mean scores given to each proposal: Fund without further discussion (F in Figure 1), Discuss in panel meeting (D), and Reject (R). Panel members could request that applications in the F or R group are reallocated to D and discussed. In a second step, the D proposals were discussed in the FTF panel meeting, ranked and funded or rejected. Random Selection (RS in Figure 1) could be used to fund or reject proposals of similar quality close to the funding threshold if the panel could not reach a decision. Funding decisions were based on the standard two-stage method, which included FTF panel meetings (TPM).

For peer review only - http://bmjopen.bmj.com/site/about/guidelines.xhtml

The simulated alternative procedure consisted only of the first step, i.e., was entirely based on the ranking of proposals based on the expert reviews: the Expert Review-Based (ERB) evaluation. In addition to the two panel members, a third expert reviewer who was not a member of the panel assessed the proposal. The same 6-point scale was used. The proposals were then allocated to one of three groups based on the mean scores (F, RS, and R in Figure 1). Random selection was used whenever the funding line went through a group of two or more applications with identical scores. The funding rate of the TPM was applied to the simulated ERB method.

Data analysis

To determine the agreement between the two evaluation methods, we used 2x2 contingency tables. We calculated the simple agreement with 95% confidence intervals (CI), which were generated using a bootstrap algorithm [5]. We also examined the agreement between the TPM and the ERB approach using only the assessments from the two panel members, thus excluding the assessment from the third reviewer. We calculated discipline- and gender-specific levels of agreement. We used chi-squared tests for categorical data to test whether the agreement differed between these mutually exclusive groups.

Costs

We determined the costs related to the evaluation. The costs comprised expenses related to the scientific assessment of the individual applications and the panel meetings. The SNSF compensates panel reviewers with USD 275 per scientific assessment. Panel reviewers further receive a meeting allowance of up to USD 550 depending on the duration of the meeting. Further, the SNSF reimburses travel expenses and accommodation costs. The five panels included 96 members and met twice in 2019.

Ethics approval

The Ethics Committee of the Canton of Berne confirmed that the study does not fall under the Federal Act on Research involving Human Beings. No reviewer, applicant or application can be identified from this study.

For peer review only - http://bmjopen.bmj.com/site/about/guidelines.xhtml
For peer review only

Patient and Public Involvement

This analysis was based on expert review reports submitted to the Swiss National Science Foundation. No patients were involved in developing the research question, outcome measures and overall design of the study.

Data availability statement

Data are available upon reasonable request. The data analyzed in this study are available to others on request after signing a data sharing agreement.

For peer review only

For peer review only - http://bmjopen.bmj.com/site/about/guidelines.xhtml

Results

Study sample and success rates

The sample consisted of 134 applications, including 124 fellowship applications and ten requests for a return grant. The mean age of applicants was 32.7 years (SD 3.2 years) in men and 33.5 years (SD 2.8 years) in women. Each reviewer received a mean of 2.5 (SD 1.4) applications to evaluate.

Table 1 shows the summary statistics of applications and success rates across disciplines, genders and the three evaluation methods: the legally binding TPM format and the simulated ERB evaluations with three or two reviewers. Most applications came from biology, followed by the STEM disciplines and the social sciences. Almost two-thirds of applications came from men. With TPM, success rates were slightly higher in women (60.4%) than in men (50.0%). This was driven by the middle group of applications that were discussed in the panels, where the success rates of women overall was 66.7% (24 of 67 applicants were women in this group). Success rates were similar across disciplines, ranging from 56.2% in the humanities to 52.2% in the social sciences. By design, overall success rates were the same with the ERB evaluations; however, the difference between genders was smaller with ERB than with TPM (Table 1).

Agreement between evaluation by ERB or TPM

Comparing the ERB evaluation based on three reviewers with the standard TPM format, the agreement overall was 80.6% (95% CI 73.9-87.3). The agreement was highest in the Medicine panel (90.0%; CI 75-100), and lowest in the Social Sciences panel (73.9%; CI 56.5-91.3). However, the statistical evidence for differences in agreement between panels was weak (P=0.58, Table 2). As expected, the agreement was higher when comparing the ERB evaluation based on the two panel members with TPM. Overall, for two reviews, the agreement was 86.6% (95% CI 80.6-91.8). It ranged from 75.0% (CI 50.0-93.8) in the Humanities panel to 91.3% (CI 78.3-100) in the Social Sciences panel. Again, there was no evidence for differences in agreement between panels (P=0.51). Both for ERB evaluation with three and two reviewers, the agreement was slightly higher for women than for men (P>0.70, Table 3).

In Table 4, we calculated agreement separately for the triage categories: Fund (F), Discuss (D), Reject (R). With the ERB evaluation based on three reviewers, agreements for F

For peer review only - http://bmjopen.bmj.com/site/about/guidelines.xhtml
For peer review only

and R were close to 100% (97.3% and 96.7%, respectively) but considerably lower for D: 64.2% (95% CI 52.2-76.1), with $P<0.001$ for differences in agreement across categories. For ERB evaluation with two reviewers (the two panel members), the agreement was 100% for F and R, but 73.1% (95% CI 62.7- 83.6) for D, with $P<0.001$ for differences in agreement.

Random selection in TPM and ERB evaluation

With the standard TPM evaluation, only eight (11.9%) of the 67 applicants in the D group, or eight (6.0%) of 134 applicants were entered into a lottery of whom four were funded. With the simulated ERB evaluation based on three reviewers, 19 (14.2%) of the 134 applicants would have entered the lottery, and with the ERB with two reviewers 23 (17.2%) applications would have been subjected to random selection.

Cost and time savings

We determined the resources that could be saved with the use of an ERB evaluation compared to the TPM. By comparison with the current valid TPM evaluation procedure for the Postdoc.Mobility, we calculated that about USD 91,000 related to the holding of meetings could have been saved if an ERB evaluation had been used for the two Postdoc.Mobility calls in 2019. This saving corresponds to 55% of total costs. Moreover, the holding of all panel sessions in 2019 amounted to 31 meeting hours, a significant workload that could have been avoided using the ERB approach. Lastly, the funding decisions could have been communicated at least one month earlier with ERB, reducing the time to notification by about 20% compared to TPM.

For peer review only - http://bmjopen.bmj.com/site/about/guidelines.xhtml

Discussion

In this comparative study of the evaluation of early-career funding applications, we found that the simulated funding outcomes of a simplified, expert review-based (ERB) approach agreed well with the official funding outcomes based on the standard, time-tested triage and panel meeting (TPM) format. Applications for fellowships covered a wide range of disciplines, from the humanities and social sciences to STEM, biology and medicine. The agreement was very high for proposals which, in the TPM evaluation, were either allocated to the Fund or Reject categories, but lower in the middle category of proposals that were discussed by the panels. More applicants entered the lottery with the simplified ERB approach than with TPM evaluation. Finally, the simplified ERB evaluation approach was associated with a substantial reduction in costs. Overall, our results support the notion that a sound evaluation of early-career funding applications is possible with an ERB approach.

Although panel review is considered as a "de facto" standard, the consistency of decisions from panels has been shown to be limited. For example, previous work by Cole [17], Hodgson [18], Fogelholm [11] and Clarke [19] found an agreement of 65% to 83% between two independent panels evaluating the same set of applications. Thus, in these studies, the funding outcome also depended on the panel that evaluated the application, and not only on the scientific content. Against this background, the agreement of over 80% between ERB and TPM in this study is remarkable. Among the different discipline-specific review panels, our results showed a slightly lower agreement in the humanities and social sciences compared to life sciences and medicine. These differences did not reach conventional levels of statistical significance but were in line with previous findings reported by Pina et al. [13].

In the middle group of applications based on the triage step of TPM, the agreement was lower; 64% with three reviewers and 73% with the two reviewers. This is not surprising considering the results from previous studies that suggest that peer review has difficulties in discriminating between applications that are neither clearly excellent nor clearly noncompetitive [20–22]. Agreement between ERB and TPM was also generally lower with ERB using three reviewers than with ERB with two reviewers. An additional reviewer may introduce a different viewpoint. Also, the third reviewer was not a member of the corresponding panel, and not involved in previous panel discussions, which have led to some degree of calibration between assessments of panel members. Such calibration is more difficult to achieve with a remote, ERB approach. However, information and briefing sessions

For peer review only - http://bmjopen.bmj.com/site/about/guidelines.xhtml

could be held to compensate for the lack of FTF panel meetings. Of note, previous studies reported that reviewers appreciated the social aspects and the camaraderie in FTF settings and that physical meetings are important for building trust among the evaluators [8,9].

We found that the panel discussions in the TPM format resulted in higher success rates for women compared to the ERB format. Gender equality is a key concern at the SNSF, which is committed to promoting women in research. The panels will have been aware of the under-representation of female researchers in certain areas, for example, the STEM disciplines, and the SNSF's agenda to promote women. It is, therefore, possible that during the panel deliberations and for funding decisions, the gender of applicants was taken into account in addition to the quality of the proposal.

We estimated that about USD 91,000 could have been saved for the two Postdoc.Mobility calls in 2019 if they had been evaluated by ERB rather than by TPM. The meeting costs represented about 55% of the total evaluation costs. In other words, the ERB evaluation based on the two panel reviewers would have cut the expenses by more than half. The experience described here with the junior Postdoc.Mobility fellowship scheme indicates that substantial cost savings could also result from simplifications in the evaluation of other funding instruments at the SNSF. Also, ERB would have reduced the time to communication of funding decisions, and unsuccessful applicants could thus have planned their next steps earlier. However, any such changes need to be considered carefully. The quality of the evaluation should not be allowed to be compromised because costs may be reduced.

To the best of our knowledge, the Health Research Council of New Zealand (HRC-NZ) [23], the Volkswagen Foundation [24], and recently the Austrian Research Fund FWF [25] are the only funders that have used or examined the use of a random selection element in the evaluation process of funding instruments, with a focus on transformative research or unconventional research ideas. The random selection for decisions on applications close to the funding threshold could avoid bias if evaluation criteria do not allow any further differentiation for a small set of similarly qualified applications [22,26]. The applicants were informed about the possible random selection and the evaluation process thus complied with the San Francisco Declaration on Research Assessment (DORA) [27], which states that funders must be explicit about assessment criteria. In this context, evaluation criteria could be weighted, or additional strategic criteria be used in the selection process if defined a priori and communicated to applicants. However, weighting or additional criteria could also lead to tied applications and thus require a lottery decision. There was some reservation on the random selection approach among some panel members, but acceptance grew over time. Of note,

For peer review only - http://bmjopen.bmj.com/site/about/guidelines.xhtml

panels applied the random selection only in a few cases, in eight (6.0%) of 134 applications. In the context of the Explorer Grant scheme of the HRC-NZ, Liu et. al [28] recently reported that most applicants agreed with the use of a random selection. In this study, the SNSF received a few unsolicited questions about the procedure but otherwise no negative or positive reactions to the use of random selection were received from applicants.

Our study has several limitations. It addressed the specific context of the SNSF Postdoc.Mobility funding scheme and results may not be generalizable to other funding instruments. The sample size was relatively small, and the study lacked statistical power, for example, to examine differences in agreement between TPM and ERB evaluation across disciplines. The two evaluation methods were not independent, since the two assessments of the panel reviewers were used for both methods. We were relying on reviewer evaluation scores which might not always perfectly reflect the quality of the proposed project, might be biased, and depend on the reviewers' previous experience with grant evaluation. However, our study design allowed us to investigate the impact of panel meetings on funding outcomes compared to an ERB approach. This study provides further insights into peer review and a modified lottery approach selection in the context of the evaluation of fellowship applications. More research on the limitations inherent in peer review and grant evaluation is urgently needed. Funders should be creative when investigating the merit of different evaluation strategies [29].

Conclusions

In conclusion, we simulated an ERB approach in the evaluation of the junior Postdoc.Mobility funding scheme at the SNSF and compared the funding outcome to the standard TPM format, which has been in use for many years. We found an overall high agreement between the two methods. Discrepancies were mainly observed in the middle group of applications that were discussed in the panel meetings. Based on the evidence that peer review has difficulties in making fine-grained differentiations between meritorious applications [20–22], we are unsure which method performs better. Our findings indicate that the ERB approach represents a viable evaluation method for the Postdoc.Mobility selection process that could save cost and time which could be invested in science and research.

For peer review only - http://bmjopen.bmj.com/site/about/guidelines.xhtml
For peer review only

Acknowledgements

We thank the Management of the SNSF Administrative Offices for helpful comments on the design of this study. We also thank the SNSF Postdoc.Mobility staff of the Administrative Offices for their excellent support in implementing the additional reviewers used for the study.

Contributors

Conceived and designed the experiments: MB KR ME. Performed the experiments: MB KR. Analyzed the data: KR RH. Contributed reagents/materials/analysis tools: MB KR ME RH. Wrote the initial draft: MB. Contributed to writing: KR ME RH.

Funding

This research was supported by institutional resources of the SNSF. ME was supported by a special project grant (grant No. 189498).

Competing interests

None declared.

For peer review only

For peer review only - http://bmjopen.bmj.com/site/about/guidelines.xhtml

References

1 Guthrie S, Ghiga I, Wooding S. What do we know about grant peer review in the health sciences? *F1000Res* 2018;**6**:1335. doi:10.12688/f1000research.11917.2

2 Guthrie S, Rodriguez Rincon D, McInroy G, *et al.* Measuring bias, burden and conservatism in research funding processes. *F1000Res* 2019;**8**:851. doi:10.12688/f1000research.19156.1

3 Snell RR. Menage a Quoi? Optimal Number of Peer Reviewers. *PLoS ONE* 2015;**10**:e0120838. doi:10.1371/journal.pone.0120838

4 Graves N, Barnett AG, Clarke P. Funding grant proposals for scientific research: retrospective analysis of scores by members of grant review panel. *BMJ* 2011;**343**:d4797–d4797. doi:10.1136/bmj.d4797

5 Herbert DL, Graves N, Clarke P, *et al.* Using simplified peer review processes to fund research: a prospective study. *BMJ Open* 2015;**5**:e008380. doi:10.1136/bmjopen-2015-008380

6 Bohannon J. Meeting for Peer Review at a Resort That's Virtually Free. *Science* 2011;**331**:27–27. doi:10.1126/science.331.6013.27

7 Gallo SA, Carpenter AS, Glisson SR. Teleconference versus Face-to-Face Scientific Peer Review of Grant Application: Effects on Review Outcomes. *PLoS ONE* 2013;**8**:e71693. doi:10.1371/journal.pone.0071693

8 Carpenter AS, Sullivan JH, Deshmukh A, *et al.* A retrospective analysis of the effect of discussion in teleconference and face-to-face scientific peer-review panels. *BMJ Open* 2015;**5**:e009138. doi:10.1136/bmjopen-2015-009138

9 Pier EL, Raclaw J, Nathan MJ, *et al.* Studying the Study Section: How Group Decision Making in Person and via Videoconferencing Affects the Grant Peer Review Process. 2015.https://wcer.wisc.edu/docs/working-papers/Working_Paper_No_2015_06.pdf

10 Vo NM, Quiggle GM, Wadhwani K. Comparative outcomes of face-to-face and virtual review meetings. *International Journal of Surgery Open* 2016;**4**:38–41. doi:10.1016/j.ijso.2016.07.002

11 Fogelholm M, Leppinen S, Auvinen A, *et al.* Panel discussion does not improve reliability of peer review for medical research grant proposals. *Journal of Clinical Epidemiology* 2012;**65**:47–52. doi:10.1016/j.jclinepi.2011.05.001

12 Obrecht M, Tibelius K, D'Aloisio G. Examining the value added by committee discussion in the review of applications for research awards. *res eval* 2007;**16**:70–91. doi:10.3152/095820207X223785

13 Pina DG, Hren D, Marušić A. Peer Review Evaluation Process of Marie Curie Actions under EU's Seventh Framework Programme for Research. *PLoS ONE* 2015;**10**:e0130753. doi:10.1371/journal.pone.0130753

For peer review only - http://bmjopen.bmj.com/site/about/guidelines.xhtml

14 Vener KJ, Feuer EJ, Gorelic L. A statistical model validating triage for the peer review process: keeping the competitive applications in the review pipeline [1]. *FASEB j* 1993;**7**:1312–9. doi:10.1096/fasebj.7.14.8224604

15 Bornmann L, Mutz R, Daniel H-D. Latent Markov modeling applied to grant peer review. *Journal of Informetrics* 2008;**2**:217–28. doi:10.1016/j.joi.2008.05.003

16 SNSF Postdoc.Mobility Regulations. http://www.snf.ch/SiteCollectionDocuments/Reglement_PM_ab2021_en.pdf

17 Cole S, Cole, Simon G. Chance and consensus in peer review. *Science* 1981;**214**:881–6. doi:10.1126/science.7302566

18 Hodgson C. How reliable is peer review? An examination of operating grant proposals simultaneously submitted to two similar peer review systems. *Journal of Clinical Epidemiology* 1997;**50**:1189–95. doi:10.1016/S0895-4356(97)00167-4

19 Clarke P, Herbert D, Graves N, *et al.* A randomized trial of fellowships for early career researchers finds a high reliability in funding decisions. *Journal of Clinical Epidemiology* 2016;**69**:147–51. doi:10.1016/j.jclinepi.2015.04.010

20 Scheiner SM, Bouchie LM. The predictive power of NSF reviewers and panels. *Frontiers in Ecology and the Environment* 2013;**11**:406–7. doi:10.1890/13.WB.017

21 Fang FC, Bowen A, Casadevall A. NIH peer review percentile scores are poorly predictive of grant productivity. *eLife* 2016;**5**:e13323. doi:10.7554/eLife.13323

22 Klaus B, del Alamo D. Talent Identification at the limits of Peer Review: an analysis of the EMBO Postdoctoral Fellowships Selection Process. Scientific Communication and Education 2018. doi:10.1101/481655

23 Health Research Council of New Zealand Explorer Grants. https://gateway.hrc.govt.nz/funding/researcher-initiated-proposals/2021-explorer-grants

24 Volkswagen Foundation Experiment! https://www.volkswagenstiftung.de/en/funding/our-funding-portfolio-at-a-glance/experiment

25 FWF 1000 Ideas Programme. https://www.fwf.ac.at/en/research-funding/fwf-programmes/1000-ideas-programme/

26 Fang FC, Casadevall A. Research Funding: the Case for a Modified Lottery. *mBio* 2016;**7**:e00422-16, /mbio/7/2/e00422-16.atom. doi:10.1128/mBio.00422-16

27 San Francisco Declaration on Research Assessment (DORA). https://sfdora.org/

28 Liu M, Choy V, Clarke P, *et al.* The acceptability of using a lottery to allocate research funding: a survey of applicants. *Res Integr Peer Rev* 2020;**5**:3. doi:10.1186/s41073-019-0089-z

For peer review only

29 Severin A, Egger M. Research on research funding: an imperative for science and society. *Br J Sports Med* 2020;:bjsports-2020-103340. doi:10.1136/bjsports-2020-103340

For peer review only

For peer review only - http://bmjopen.bmj.com/site/about/guidelines.xhtml

Figures and Tables

Figure 1. Design of the study comparing the Expert Review-Based evaluation (ERB) with the Triage-Panel Meeting (TPM) format. The ERB and the TPM were dependent in terms of the two assigned panel reviewers per application. The third reviewers were only added for the ERB, their assessments were not considered for the TPM and therefore the official funding outcome.

For peer review only

Table 1. Success rates by gender of applicants, by discipline and type of evaluation.

	All applicants		Women		Men	
**Discipline**	**N**	**N funded (%)**	**N**	**N funded (%)**	**N**	**N funded (%)**
**TPM**						
All disciplines	134	72 (53.7)	48	29 (60.4)	86	43 (50.0)
Humanities	16	9 (56.2)	9	4 (44.4)	7	5 (71.4)
Social Sciences	23	12 (52.2)	10	7 (70.0)	13	5 (38.5)
STEM	35	19 (54.3)	10	6 (60.0)	25	13 (52.0)
Biology	40	21 (52.5)	14	8 (57.1)	26	13 (50.0)
Medicine	20	11 (55.0)	5	4 (80.0)	15	7 (46.7)
**ERB (3 reviewers*)**						
All disciplines	134	72 (53.7)	48	27 (56.3)	86	45 (52.3)
Humanities	16	9 (56.3)	9	5 (55.6)	7	4 (57.1)
Social Sciences	23	12 (52.2)	10	6 (60.0)	13	6 (46.2)
STEM	35	19 (54.3)	10	4 (40.0)	25	15 (60.0)
Biology	40	21 (52.5)	14	8 (57.1)	26	13 (50.0)
Medicine	20	11 (55.0)	5	4 (80.0)	15	7 (46.7)
**ERB (2 reviewers[&])**						
All disciplines	134	72 (53.7)	48	25 (52.1)	86	47 (54.7)
Humanities	16	9 (56.3)	9	5 (55.6)	7	4 (57.1)
Social Sciences	23	12 (52.2)	10	6 (60.0)	13	6 (46.2)
STEM	35	19 (54.3)	10	4 (40.0)	25	15 (60.0)
Biology	40	21 (52.5)	14	7 (50.0)	26	14 (53.8)
Medicine	20	11 (55.0)	5	3 (60.0)	15	8 (53.3)

Abbreviations: N: Number of applications; STEM: Science, Technology, Engineering, Mathematics; TPM: Triage-panel meeting format; ERB: Expert review-based evaluation.

*Two of the three expert reviewers were also members of the evaluation panel.

[&]Both expert reviewers were also members of the evaluation panel.

For peer review only - http://bmjopen.bmj.com/site/about/guidelines.xhtml

Table 2. Agreement between the simulated expert review-based (ERB) evaluation and the triage-panel meeting (TPM) format, by discipline.

Discipline	N	Funded by TPM		Agreement (%) (95% CI)
**Funded by ERB (3 reviewers*)**		Yes	No	
All disciplines	Yes	59	13	80.6
	No	13	49	(73.9-87.3)
Humanities	Yes	7	2	75.0
	No	2	5	(50.0-93.8)
Social Sciences	Yes	9	3	73.9
	No	3	8	(56.5-91.3)
STEM	Yes	15	4	77.1
	No	4	12	(62.9-91.4)
Biology	Yes	18	3	85.0
	No	3	16	(72.5-95)
Medicine	Yes	10	1	90.0
	No	1	8	(75-100)
P-value				0.58
**Funded by ERB (2 reviewers[&])**				
All disciplines	Yes	63	9	86.6
	No	9	53	(80.6-91.8)
Humanities	Yes	7	2	75.0
	No	2	5	(50.0-93.8)
Social Sciences	Yes	11	1	91.3
	No	1	10	(78.3-100)
STEM	Yes	16	3	82.9
	No	3	13	(68.6-94.3)
Biology	Yes	19	2	90.0
	No	2	17	(80-97.5)
Medicine	Yes	10	1	90.0
	No	1	8	(75-100)
P-value				0.51

Abbreviations: N: Number of applications; CI: Confidence interval; STEM: Science, Technology, Engineering, Mathematics; ERB: Expert review-based evaluation; TPM: Triage-panel meeting format.

P-values for differences in agreement across disciplines from chi-squared test.

*Two of the three expert reviewers were also members of the evaluation panel.

[&]Both expert reviewers were also members of the evaluation panel.

For peer review only - http://bmjopen.bmj.com/site/about/guidelines.xhtml

For peer review only

Table 3. Agreement between the simulated expert review-based (ERB) evaluation and the triage-panel meeting (TPM) format, by gender.

Gender		Funded by TPM		Agreement (%) (95% CI)
**Funded by ERB (3 reviewers*)**		Yes	No	
Women	Yes	24	3	83.3
	No	5	16	(72.9-93.8)
Men	Yes	35	10	79.1
	No	8	33	(69.8-87.2)
P-value				0.71
**Funded by ERB (2 reviewers[&])**				
Women	Yes	24	1	87.5
	No	5	18	(77.1-95.8)
Men	Yes	39	8	86.0
	No	4	35	(77.9-93.0)
P-value				0.99

Abbreviations: N: Number of applications; CI: Confidence interval; STEM: Science, Technology, Engineering, Mathematics; ERB: Expert review-based evaluation; TPM: Triage-panel meeting format.

P-values for differences in agreement across genders from chi-squared test.

*Two of the three expert reviewers were also members of the evaluation panel.

[&]Both expert reviewers were also members of the evaluation panel.

For peer review only - http://bmjopen.bmj.com/site/about/guidelines.xhtml

Table 4. Agreement between the simulated expert review-based (ERB) evaluation and the triage-panel meeting (TPM) format, by triage results.

**Triage result**		**Funded by TPM**		**Agreement (%) (95% CI)**
**Funded by ERB (3 reviewers*)**		Yes	No	
Fund (F)	Yes	36	0	97.3
	No	1	0	(91.9-100)
Discuss (D)	Yes	23	12	64.2
	No	12	20	(52.2-76.1)
Reject (R)	Yes	0	1	96.7
	No	0	29	(90.0-100)
P-value				<0.001
**Funded by ERB (2 reviewers[&])**				
Fund (F)	Yes	37	0	100
	No	0	0	
Discuss (D)	Yes	26	9	73.1
	No	9	23	(62.7-83.6)
Reject (R)	Yes	0	0	100
	No	0	30	
P-value				<0.001

Abbreviations: N: Number of applications; CI: Confidence interval; STEM: Science, Technology, Engineering, Mathematics; ERB: Expert review-based evaluation; TPM: Triage-panel meeting format.

P-values for differences in agreement across triage groups from chi-squared test.

*Two of the three expert reviewers were also members of the evaluation panel.

[&]Both expert reviewers were also members of the evaluation panel.

For peer review only

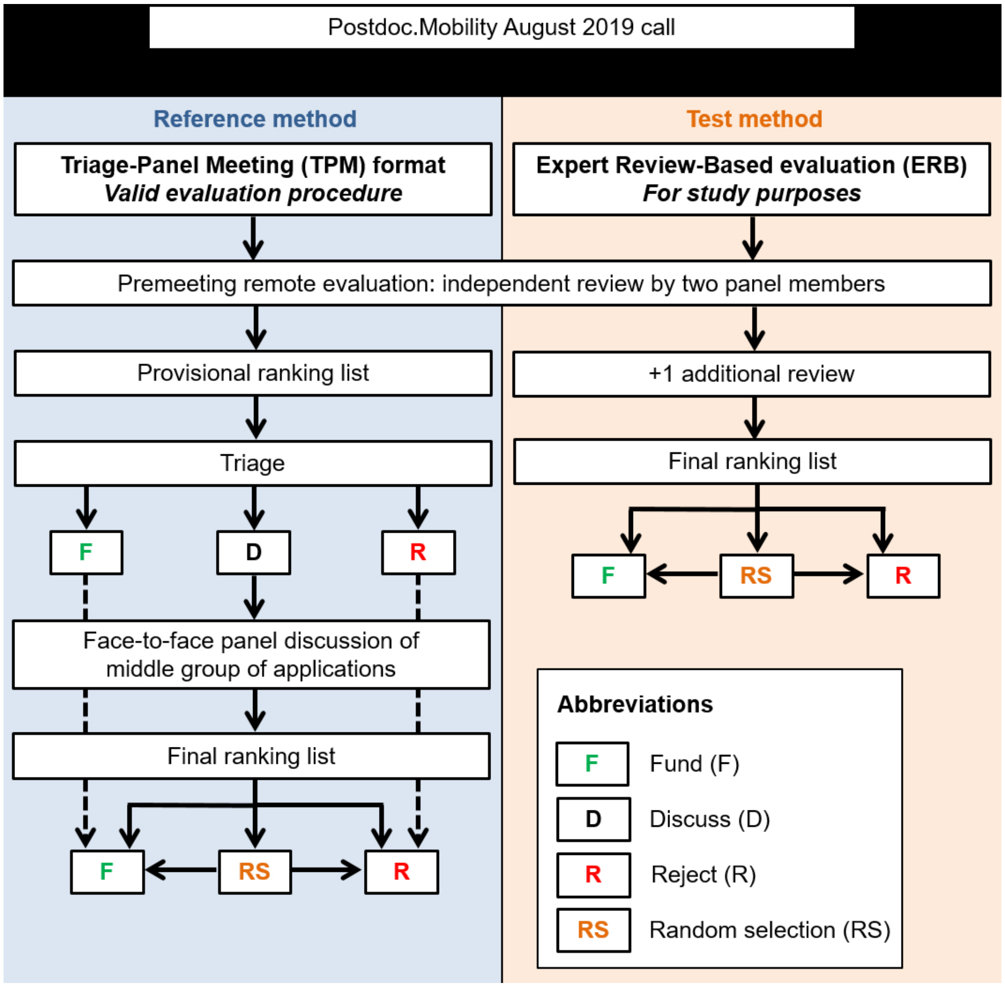

Figure 1. Design of the study comparing the Expert Review-Based evaluation (ERB) with the Triage-Panel Meeting (TPM) format. The ERB and the TPM were dependent in terms of the two assigned panel reviewers per application. The third reviewers were only added for the ERB, their assessments were not considered for the TPM and therefore the official funding outcome.
